# Design Strategies to Target Joint Resident Mesenchymal Stem Cells for Osteochondral Regeneration

**DOI:** 10.3390/cells15141290

**Published:** 2026-07-18

**Authors:** Khan Sharun, Shajahan Amitha Banu, Sathish Muthu, Cristian Pablo Pennisi

**Affiliations:** 1 Regenerative Medicine Group, Department of Health Science and Technology, Aalborg University, 9260 Gistrup, Denmark; amithabs@hst.aau.dk; 2 Orthopaedic Research Group, Department of Spine Surgery, Coimbatore 641045, India; drsathishmuthu@gmail.com; 3 Central Research Laboratory, Aarupadai Veedu Medical College and Hospital, Vinayaka Mission’s Research Foundation, Puducherry 607403, India

**Keywords:** cartilage regeneration, bone tissue engineering, regenerative medicine, stem cell therapy, osteoarthritis, tissue engineering

## Abstract

Restoration of the osteochondral unit remains a major challenge in regenerative orthopaedics, largely due to the limited intrinsic healing capacity of articular cartilage and the complex, multilayered nature of the cartilage–bone interface. Osteochondral regeneration must accommodate differences in cellular composition, vascularization, metabolic demand, and mechanical properties between cartilage and bone, while simultaneously recreating a stable, functional interface. While exogenous mesenchymal stem cell (MSC) therapies have dominated the field, their clinical translation has been hindered by donor variability, phenotypic instability, logistical complexity, and inconsistent long-term outcomes. Resident stem cells from sources such as articular cartilage, bone marrow, periosteum, synovium, synovial fluid, and adipose tissue (infrapatellar fat pad) can act as potential targets for in situ osteochondral regenerative therapies. Joint-resident MSCs are adapted to the biomechanical and biochemical environment of the joint and may therefore represent a promising cell source for osteochondral regeneration; however, much of the supporting evidence remains preclinical. Effective osteochondral repair depends on the precise orchestration of stem cell recruitment, maintenance of chondrogenic phenotypes, induction of osteogenic differentiation in the subchondral compartment, and modulation of local immune responses. Patient-specific factors, including age, inflammatory status, and the severity of osteoarthritis, can significantly influence the regenerative potential of resident MSC populations and should therefore guide biomaterial design strategies. The proposed niche-by-design framework integrates stem cell biology with advanced biomaterial engineering, offering a rational roadmap for developing next-generation therapies that promote endogenous osteochondral regeneration through targeted activation of joint-resident progenitor cells.

## 1. Introduction

The quest to restore damaged articular cartilage and subchondral bone remains one of the most persistent challenges in regenerative orthopaedics [1]. Articular cartilage has limited intrinsic healing potential due to its avascular, aneural, and alymphatic nature [2]. The osteochondral unit is a complex biomechanical and biochemical continuum that requires the coordinated regeneration of multiple tissues. Effective repair, therefore, requires coordinated regeneration of both cartilage and subchondral bone within a spatially organized microenvironment. Over the past two decades, exogenous mesenchymal stem cell (MSC)-based therapies have dominated osteochondral regeneration strategies [3]. While early preclinical and clinical studies were encouraging, achieving consistent long-term outcomes has been difficult [4]. Challenges such as donor variability, phenotypic instability during expansion, risks of undesired differentiation, regulatory complexity, and the logistical burden of cell preparation have limited the scalability and predictability of transplantation-based approaches [3,5]. As these limitations have become increasingly apparent, attention has shifted toward alternative strategies that leverage endogenous repair mechanisms rather than relying solely on cell delivery.

Joint-resident stem and progenitor cell populations located within articular cartilage, bone marrow, periosteum, synovium, synovial fluid, and the infrapatellar fat pad have emerged as promising therapeutic targets [6,7]. These cells are inherently adapted to the joint’s biomechanical loading patterns, biochemical milieu, and inflammatory environment [8]. Targeting such resident MSC niches offers several conceptual advantages: reduced need for cell harvesting and expansion, improved biological compatibility, and the potential for more seamless tissue integration [7]. Importantly, advances in single-cell transcriptomics, lineage tracing, and niche biology have recently improved our understanding of the heterogeneity, spatial distribution, and functional specialization of these joint-resident populations [9]. This growing biological insight makes it increasingly feasible to design interventions that selectively mobilize and instruct endogenous cells in situ.

Clinically, the concept of recruiting resident MSCs is not new. Bone marrow stimulation techniques, such as microfracture or subchondral drilling, aim to release bone marrow-derived MSCs into defect sites [10]. Although these approaches often provide short-term symptomatic relief, the resulting repair tissue is frequently fibrocartilaginous and biomechanically inferior, leading to long-term degeneration [11,12]. These outcomes highlight a critical limitation: recruitment alone is insufficient without appropriate spatial and biochemical guidance. This realization has catalyzed a paradigm shift. Rather than simply delivering cells or relying on passive marrow stimulation, contemporary strategies increasingly focus on engineering biomaterials that actively target resident MSC niches, enhancing recruitment, directing lineage specification, and promoting organized osteochondral regeneration [13,14,15]. The convergence of improved niche biology, advanced biomaterial design, and growing clinical demand for scalable therapies makes this an opportune moment to reframe osteochondral repair around the deliberate orchestration of joint-resident MSC populations.

Despite numerous reviews on osteochondral scaffolds and MSC-based therapies, a critical gap remains in integrating the biology of joint-resident MSC niches with rational biomaterial design principles to achieve effective in situ osteochondral regeneration. Most existing reviews catalogue scaffold compositions, growth factors, or cell delivery strategies, yet few synthesize how endogenous MSC populations can be systematically targeted through spatially and temporally programmed design cues. In this narrative review, we address this unmet need by examining how joint-resident MSC reservoirs contribute to repair and proposing a design-oriented framework that aligns with biological objectives. By reframing osteochondral regeneration as a problem of niche-targeted orchestration rather than scaffold supplementation alone, this review aims to provide a structured perspective to guide future biomaterial development toward predictable, translationally relevant in situ regeneration.

## 2. Exogenous Mesenchymal Stem Cell Therapy

Exogenous MSC therapy has attracted significant attention as a regenerative approach for the repair of osteochondral defects [16]. Early investigative and translational efforts have focused mainly on isolating joint-resident stem cells, followed by ex vivo expansion under controlled culture conditions to achieve therapeutically relevant cell numbers [17]. Subsequently, the expanded cells are reintroduced into osteochondral defects in combination with biomaterial scaffolds designed to support cell survival, retention, and differentiation [17,18]. These scaffold-assisted, cell-based strategies aim to recreate key aspects of the native osteochondral microenvironment by providing structural support and instructive biochemical cues that guide tissue formation [19]. While this approach has demonstrated encouraging regenerative outcomes, it has also highlighted the need for more refined strategies that can efficiently harness resident stem cell populations directly within the joint, reducing the reliance on cell harvesting and transplantation while preserving the inherent advantages of endogenous repair mechanisms [20].

Exogenous MSC therapies present several important disadvantages and safety considerations that continue to limit their widespread clinical translation. One of the primary challenges is the significant heterogeneity and variability in the potency of MSC products [21]. MSC populations can differ markedly depending on their tissue source, donor age and health status, as well as the methods used for cell isolation, expansion, and cryopreservation [22,23]. These factors can substantially alter the cellular phenotype, secretory profile, and regenerative potential of MSCs, making it difficult to standardize therapies, compare outcomes across studies, or reliably predict their in vivo behaviour [22]. In addition, culture-associated risks arise during the ex vivo expansion process required to generate clinically relevant cell numbers [24]. Prolonged in vitro culture can lead to cellular senescence, genetic instability, or chromosomal abnormalities, and may alter the differentiation capacity of MSCs [24,25]. Furthermore, manufacturing-related issues such as contamination, variability in culture reagents, or inconsistencies in processing protocols have raised safety concerns in some cell therapy programs [26]. An additional concern is the use of animal-derived culture supplements, such as fetal bovine serum, which may introduce batch variability, xenogeneic contaminants, immunogenic risks, and additional regulatory challenges [27].

Although MSC therapies are generally considered relatively safe, adverse events have been reported in clinical studies, highlighting the need for continued vigilance [28]. While severe acute immune reactions are uncommon, safety summaries from clinical trials have reported complications including thromboembolic events, fibrosis, and ectopic tissue formation, underscoring the importance of rigorous long-term safety monitoring [29,30]. These potential risks also underscore the necessity of transparent patient counselling and informed consent regarding the still-uncertain long-term outcomes of MSC-based interventions [30]. Another important limitation relates to uncertainty surrounding the mechanisms of action of transplanted MSCs. Increasing evidence suggests that the therapeutic benefits of MSCs may primarily arise from paracrine signalling and the secretion of bioactive factors rather than durable engraftment and differentiation into cartilage or bone tissue [31,32]. If MSCs act mainly through transient signalling effects, determining optimal dosing, delivery routes, and treatment frequency becomes more complex.

Regulatory pathways for MSC products vary by jurisdiction and by whether a product is autologous, minimally manipulated, or presented as an allogeneic, expanded cell therapy categories that affect whether a therapy is regulated as a tissue, a medicinal product, or an advanced therapy medicinal product (ATMP) [33,34]. Regulators require robust GMP manufacturing, validated potency assays, sterility and identity testing, donor screening, and well-defined release criteria; yet there is no universally accepted potency readout that predicts clinical performance for osteochondral repair [35,36]. These issues, together with batch-to-batch variability and the high cost of GMP production and controlled clinical trials, slow translation and commercial adoption [35].

While exogenous MSC therapy focuses on delivering externally expanded cells to the defect site, an emerging regenerative paradigm seeks to activate or recruit endogenous stem/progenitor cells that already reside within the joint microenvironment [7,8,37]. One of the major advantages of resident stem cell-based strategies is that they avoid several of the manufacturing and regulatory challenges associated with exogenous MSC therapies. Because endogenous cells are not harvested, expanded, or manipulated outside the body, these approaches typically bypass the need for complex GMP cell production and reduce the risks of contamination, genetic instability, or phenotypic drift that may arise during in vitro expansion.

## 3. Joint Resident Stem Cells

**Figure 1:**
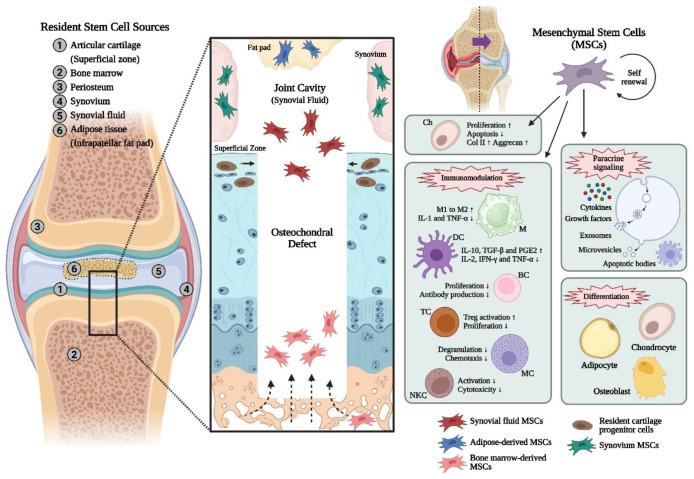
Resident mesenchymal stem cells for osteochondral regeneration. The figure illustrates the anatomical distribution and potential functional mechanisms of resident mesenchymal stem cells (MSCs) in osteochondral regeneration. The major endogenous MSC reservoirs include (1) articular cartilage (superficial zone), (2) bone marrow, (3) periosteum, (4) synovium, (5) synovial fluid, and (6) adipose tissue (e.g., infrapatellar fat pad). The right panel summarizes the key biological functions of MSCs that contribute to tissue regeneration. These include differentiation, paracrine signalling through cytokines, growth factors, exosomes, microvesicles, and apoptotic bodies, as well as immunomodulatory activities. ↑: Increased, ↓: Decreased, Ch: Chondrocyte, M: Macrophage, DC: Dendritic cells, BC: B cell, TC: T cell, MC: Mast cell, and NKC: Natural killer cell. Created with BioRender.com.

Resident stem cell populations within the joint microenvironment play a central role in osteochondral regeneration by serving as locally available progenitors that respond to injury and contribute to the restoration of both cartilage and subchondral bone (Figure 1) [8]. Among these, MSCs residing in the synovial membrane and synovial fluid have emerged as particularly important because they not only increase in number following joint damage but also display strong chondrogenic potential [38,39]. These synovium-derived cells are multipotent, exhibit superior chondrogenic differentiation compared with other joint-derived MSCs, and migrate to lesion sites, making them among the most biologically active resident cell pools in cartilage regeneration [39]. Similarly, the superficial zone of articular cartilage contains a population of slow-cycling progenitor cells capable of participating in tissue homeostasis and repair [6,40]. These superficial zone progenitors can modulate cartilage matrix synthesis and maintain surface integrity, suggesting an intrinsic repair mechanism that becomes particularly relevant when injury disrupts the structural barrier between cartilage layers [40]. Although their regenerative potential may be hindered in deep focal defects or when the tidemark (boundary between calcified and non-calcified cartilage) is breached, they remain critical contributors to the early cellular response after mild or superficial injuries.

The subchondral bone marrow represents another essential reservoir of endogenous stem cells. BM-MSCs are widely recognized for their capacity to differentiate into chondrocytes and osteoblasts [15,41]. Microfracture-induced repair relies on the recruitment of these marrow-resident progenitors into the defect space, where they generate a fibrocartilage repair tissue that partially restores joint function [42,43]. While fibrocartilage lacks the biomechanical durability of native hyaline cartilage, the process underscores the importance of marrow-derived MSCs as a natural fallback mechanism for joint repair [42]. In addition to marrow spaces, the periosteum functions as an active stem-cell niche containing progenitor cells with strong chondrogenic and osteogenic potential [44]. These cells can differentiate toward cartilage under low-oxygen or mechanically constrained conditions and toward bone under more vascularized environments, making periosteal MSCs key participants in osteochondral junction healing, where both tissues need to be regenerated simultaneously.

The infrapatellar fat pad (IFP) is another joint-resident reservoir of MSCs with potent immunomodulatory and chondrogenic properties [45]. It represents a particularly relevant MSC reservoir because of its anatomical proximity to the synovium, articular cartilage, and patellofemoral joint space. IFP-derived MSCs combine features of adipose-derived MSCs with adaptation to the intra-articular environment. Although not as extensively detailed as synovial or bone marrow sources, IFP-derived MSCs are a relevant endogenous pool that contributes to joint homeostasis and participates in tissue repair under inflammatory conditions by secreting trophic and anti-catabolic mediators that influence cartilage regeneration [46]. Their location within the joint also makes them clinically accessible during arthroscopic or open knee procedures, supporting their potential role in one-stage regenerative strategies [45]. The biological relevance of IFP-MSCs is not limited to their differentiation capacity. These cells can secrete trophic, anti-inflammatory, and anti-catabolic mediators that may influence synovial inflammation, cartilage matrix turnover, and local progenitor cell activity [46]. This is particularly important in osteoarthritis, where the IFP is not a passive fat depot but an active immunometabolic tissue that can contribute to either joint inflammation or repair, depending on the disease context. Therefore, IFP-MSCs should be considered both as potential regenerative effectors and as cells whose function may be altered by inflammatory and degenerative joint environments [47].

Meanwhile, cartilage-resident stem or progenitor cells, although fewer in number, are increasingly recognized as regulators of matrix turnover [6]. Their presence within the articular cartilage matrix suggests that the tissue retains a limited but significant intrinsic regenerative potential. These cartilage-resident progenitors become activated in response to biochemical cues and mechanical disruption, contributing directly to matrix deposition and potentially facilitating the integration of repair tissue into the native cartilage structure [8]. The collective behaviour of these resident stem cell populations is influenced by the complex biomechanical and biochemical environment of the osteochondral unit [6,8]. Injury triggers a cascade of events that includes the release of growth factors, recruitment signals, inflammatory mediators, and matrix breakdown products, all of which act to mobilize endogenous MSCs [6].

The relative contribution of each source depends on the depth of injury, size of the defect, vascular involvement, and the structural continuity between cartilage zones. For superficial defects, superficial zone progenitors and synovial MSCs may dominate the repair response, whereas deep or full-thickness defects rely heavily on BM-MSCs and periosteal progenitors [8,48]. It is already well known that the endogenous MSCs do not function solely through direct differentiation. Their paracrine actions are equally important, as they modulate inflammation, recruit additional cell populations, and regulate the synthesis and remodelling of the extracellular matrix [49]. Furthermore, MSCs in particular exert strong immunomodulatory effects, dampening inflammatory responses that would otherwise accelerate cartilage degeneration (Figure 1) [50].

Collectively, these interactions demonstrate that endogenous stem cell-mediated osteochondral regeneration is a coordinated, multi-tissue process rather than an isolated event. Understanding the biology of these resident progenitor sources is essential for developing regenerative therapies that leverage or enhance intrinsic repair mechanisms [6,8]. Strategies such as biological stimulation, mechanical conditioning, microfracture augmentation, and intra-articular delivery of trophic molecules aim to activate or support these endogenous cell populations [6]. Clarifying the distinct roles of resident progenitors underscores that osteochondral regeneration depends on the coordinated activity of multiple local stem cell niches. Each niche contributes in its own complementary way to rebuilding subchondral bone and restoring the structural and functional integrity of damaged cartilage.

Although joint-resident MSCs are often discussed collectively, they should not be regarded as a homogeneous cell population [1,3]. Resident progenitors derived from synovium, synovial fluid, bone marrow, periosteum, infrapatellar fat pad, and articular cartilage differ in developmental origin, anatomical niche, baseline transcriptional state, proliferative capacity, differentiation bias, immunomodulatory function, and responsiveness to inflammatory and mechanical stimuli [3]. These differences have important implications for biomaterial design because a cue that efficiently recruits or instructs one MSC population may not produce the same response in another [3,51].

Table 1 provides a concise comparative overview of the major joint-resident MSC populations relevant to osteochondral regeneration, highlighting their proliferation capacity, chondrogenic potential, accessibility, and clinical relevance. The comparison emphasizes that no single resident MSC source is universally optimal. Synovial membrane- and synovial fluid-derived MSCs are particularly attractive for cartilage repair due to their superior migratory capacity and chondrogenic potential, whereas bone marrow-derived MSCs are most relevant for full-thickness osteochondral defects that penetrate the subchondral bone [52,53,54,55]. Cartilage-resident progenitor cells may contribute to superficial cartilage repair and tissue integration, but are limited by their low abundance and restricted migratory capacity [56,57,58]. Infrapatellar fat pad-derived MSCs are readily accessible and possess robust regenerative potential; however, their therapeutic efficacy may be influenced by the inflammatory and fibrotic status of the joint microenvironment [59,60,61]. Therefore, resident MSC-targeting strategies should be selected based on defect depth, joint inflammatory status, patient age, and the specific tissue compartment to be repaired.

## 4. Biophysical and Biological Drivers of Resident Stem Cell-Mediated Regeneration

Mechanical and biophysical cues are major regulators of resident MSC differentiation, matrix synthesis, and regenerative behavior within the osteochondral unit [67,68]. Compression, shear stress, hydrostatic pressure, and matrix stiffness all influence the differentiation pathways of resident MSCs [69]. Physiological loading conditions promote chondrogenesis, whereas abnormal or excessive loading can lead to fibrotic or hypertrophic differentiation [70]. Mechanotransduction pathways involving integrins and cytoskeletal networks regulate gene expression and matrix synthesis [71]. Therapeutic strategies aimed at optimizing joint mechanics, such as unloading protocols, osteotomy, or targeted physiotherapy, may indirectly enhance resident stem cell-mediated repair. Biomaterial scaffolds designed to transmit physiologic mechanical signals may further augment this effect when used in osteochondral defects [20,72].

At the molecular level, resident MSCs interpret joint loading through mechanotransduction pathways involving integrins, focal adhesion kinase (FAK), RhoA/ROCK-mediated cytoskeletal tension, mechanosensitive ion channels, and downstream transcriptional regulators [73,74]. YAP and TAZ are key mechanosensitive transcriptional co-regulators that respond to substrate stiffness, cell shape, and cytoskeletal tension [75]. Increased matrix stiffness and cytoskeletal contractility generally promote YAP/TAZ activation, whereas more compliant microenvironments tend to limit their activity [76]. Sustained YAP/TAZ activation under conditions of elevated mechanical tension has been associated with osteogenic, fibrogenic, and hypertrophic gene programs, whereas reduced or tightly regulated YAP/TAZ activity within compliant three-dimensional matrices is generally associated with more stable chondrogenic differentiation [77]. Mechanosensitive ion channels such as Piezo1, Piezo2, and TRPV4 provide additional routes through which compression, osmotic changes, and fluid-flow-induced forces regulate intracellular calcium signaling and downstream gene expression [78,79]. TRPV4 activation has been associated with physiological and chondroprotective mechanotransduction, contributing to cartilage matrix homeostasis, whereas dysregulated Piezo-mediated mechanosensing may contribute to inflammatory and catabolic responses under excessive mechanical loading [80]. Collectively, these findings suggest that biomaterials designed for resident MSC targeting should not only match tissue-level mechanics but also modulate cell-level mechanotransduction to promote SOX9-driven chondrogenesis while limiting expression of hypertrophic, osteogenic, and fibrotic markers, including RUNX2, COL10A1, and COL1A1 [63].

Inflammation plays a complex and context-dependent role in endogenous tissue regeneration [81]. In the acute phase, inflammatory signals can be beneficial by promoting the recruitment of progenitor cells, enhancing their proliferation, and initiating extracellular matrix remodelling processes that are essential for tissue repair [82]. These early inflammatory cues help activate dormant repair mechanisms and facilitate cellular crosstalk within the regenerative niche [83,84]. In contrast, sustained or chronic inflammation impairs cartilage homeostasis and regenerative capacity. Prolonged exposure to pro-inflammatory cytokines disrupts the chondrogenic niche, induces cellular senescence, and stimulates the production of catabolic enzymes such as matrix metalloproteinases and aggrecanases, leading to progressive degradation of the cartilage extracellular matrix [85]. In osteoarthritis, persistent synovitis results in continuous exposure of synovial MSCs to a hostile inflammatory environment, compromising their viability, altering their differentiation capacity, and ultimately limiting their contribution to effective cartilage repair [86]. Consequently, modulation of the inflammatory microenvironment has emerged as a central challenge in endogenous cartilage regeneration strategies. Approaches, including targeted biologic therapies, immunomodulatory biomaterials, and scaffolds with controlled or spatiotemporal release of anti-inflammatory and pro-regenerative factors, aim to restore a regenerative balance within the joint [81]. Accumulating evidence suggests that successful endogenous repair requires precise temporal regulation of inflammation, rather than its complete suppression, to preserve early reparative signals while preventing chronic tissue damage [87]. Interestingly, experimental studies using rat models of osteoarthritis have revealed that the number of synovial fluid MSCs correlates positively with the severity of synovitis [38]. Moreover, synovial fluid MSCs isolated from osteoarthritic joints exhibit higher expression of tissue-reparative genes compared with those derived from knees without structural joint damage [38]. These findings suggest that inflammatory joint environments may simultaneously impair regenerative outcomes while mobilizing and activating endogenous progenitor populations [67]. Harnessing this intrinsic repair response while mitigating the deleterious effects of chronic inflammation represents a critical opportunity to advance cell-free and endogenous regeneration strategies for cartilage repair.

Macrophages are central regulators of the inflammatory-regenerative balance [88]. Classically activated M1-like macrophages secrete pro-inflammatory mediators such as IL-1β, TNF-α, IL-6, reactive oxygen species, and matrix-degrading enzymes, which can amplify synovitis, impair MSC chondrogenesis, and accelerate cartilage matrix degradation [89,90]. In contrast, alternatively activated M2-like macrophages produce anti-inflammatory and pro-reparative mediators that support resolution of inflammation, extracellular matrix remodeling, angiogenic regulation, and progenitor cell-mediated tissue repair [91]. Therefore, successful endogenous osteochondral regeneration likely requires not simply suppression of inflammation, but temporal modulation of macrophage phenotype from an early debris-clearing and recruitment-supportive response toward a reparative M2-like state. Immunomodulatory biomaterials that regulate macrophage polarization may thus indirectly enhance resident MSC recruitment, survival, and lineage stabilization.

MSCs exhibit a pronounced migratory capacity, which has been linked to the expression of specific surface markers and transcriptional regulators that actively govern cell movement [92]. Among these, CD44 plays a central role. As a principal receptor for hyaluronan, CD44 is widely recognized as a defining MSC surface marker and serves as a key mediator of cell adhesion, cytoskeletal organization, and directed migration [93]. Functional studies have demonstrated that disruption of CD44 signalling, either through antibody-mediated blockade or RNA interference, leads to a substantial reduction in MSC motility, underscoring its importance in regulating migratory behaviour [94]. In contrast, enhanced CD44 expression has been shown to enhance MSC homing efficiency, particularly toward sites of inflammation, where hyaluronan-rich extracellular matrices provide directional cues for cell recruitment [95]. In addition to surface adhesion molecules, intracellular transcriptional regulators also play a role in the migratory phenotype of MSCs [92,96]. Runt-related transcription factor 2 (Runx2), best known for its role in osteogenic differentiation, is highly expressed in MSCs and has been increasingly associated with cell motility across multiple cell types [96]. Runx2 is thought to influence migration by modulating the expression of genes involved in cytoskeletal dynamics, extracellular matrix interactions, and cell signalling pathways [97]. Together, the coordinated action of migration-associated surface receptors, such as CD44, and transcriptional regulators, such as Runx2, provides a mechanistic basis for the enhanced migratory and homing capabilities of MSCs, which are critical to their therapeutic efficacy in tissue repair and regeneration.

Resident MSCs respond to gradients of transforming growth factor β (TGF-β), bone morphogenetic proteins (BMPs), fibroblast growth factor (FGF), and chemokines [19,98]. These biochemical signals act in synergy with mechanical cues to drive lineage specification. In the osteochondral unit, spatial gradients help maintain distinct yet interconnected tissue zones. Therapeutic strategies that recreate or enhance these gradients, such as the localized delivery of growth factors or biomaterials capable of forming gradients, may improve the recruitment and differentiation of resident cells [13]. However, achieving controlled and sustained gradients in vivo remains technically challenging.

Taken together, these findings highlight that resident MSC-mediated regeneration is governed by a highly integrated network of mechanical, inflammatory, migratory, and biochemical signals within the osteochondral microenvironment. Rather than acting independently, these cues dynamically interact to regulate progenitor cell recruitment, survival, lineage commitment, and tissue remodeling. Consequently, successful endogenous cartilage repair strategies will likely require multifactorial approaches that simultaneously optimize biomechanical conditions, modulate inflammatory responses, enhance cell homing, and provide spatially and temporally controlled morphogenetic signals.

## 5. Targeting Resident Stem Cells

In the context of osteochondral regeneration, biomaterials designed to mimic the extracellular matrix or provide structural support can be used to guide resident stem cells into areas of tissue defects. These scaffolds may incorporate biochemical cues, controlled mechanical properties, or topographical features that promote host cell infiltration and appropriate differentiation [99]. Strategies such as layered osteochondral scaffolds, hydrogel microenvironments, and nanofiber architectures have demonstrated potential to promote endogenous repair [13]. Small molecules and growth factors can be used to activate resident progenitors without the need for cell transplantation. Agents that modulate the Wnt, Hedgehog, or Notch pathways, as well as chemotactic cytokines, have been shown to enhance progenitor migration and chondrogenesis [49].

Resident MSC-targeting approaches differ substantially in their biological rationale, technical complexity, and translational readiness [100,101]. Marrow stimulation procedures such as microfracture and nanofracture are clinically accessible and exploit bone marrow-derived progenitor recruitment, but they often produce fibrocartilage rather than durable hyaline cartilage [42,43]. Chemokine-loaded or homing peptide-functionalized scaffolds offer more specific control over endogenous cell recruitment, yet their effectiveness depends on establishing and maintaining bioactive chemotactic gradients in the mechanically demanding and enzymatically active joint environment [102]. Growth factor- or small molecule-releasing biomaterials can provide stronger instructive signals for lineage specification, but they introduce dose-control, release-kinetic, safety, and regulatory challenges [101,103]. Platelet-derived biologics provide a clinically familiar and autologous source of trophic factors, although variability in composition and preparation limits reproducibility [104,105]. Finally, gradient and multilayered scaffolds are conceptually well suited for osteochondral regeneration because they can reproduce the cartilage–bone interface, but they require robust fixation, scalable manufacturing, and evidence of long-term functional superiority [106,107]. Therefore, future strategies should be evaluated not only by initial repair tissue formation but also by their ability to recruit the correct endogenous cells, stabilize hyaline chondrogenesis, prevent hypertrophy, integrate with host cartilage and bone, and remain practical for single-stage clinical use.

Table 2 provides a brief description of the major joint-associated resident MSC sources, their characteristic surface marker profiles [7,66,108], and potential targeting strategies for osteochondral repair.

## 6. Bioactive Factors for Osteochondral Regeneration

Bioactive factors play a central role in regulating the complex cellular and molecular events required for successful osteochondral regeneration [13,122]. The coordinated repair of cartilage and subchondral bone relies on tightly controlled signalling cues that govern stem cell recruitment, proliferation, differentiation, and matrix remodelling [123,124]. Rather than cataloguing these molecules individually, their roles can be more coherently understood within a functional design framework aligned with the biological requirements of osteochondral repair. In this context, bioactive cues, including inorganic ions, growth factors, chemokines, growth factor–rich biologics, and small-molecule drugs, serve distinct yet interconnected regenerative objectives: (1) recruitment of reparative cells, (2) lineage-specific differentiation and phenotypic stabilization, (3) spatial organization across cartilage and bone compartments, and (4) modulation of inflammation and matrix remodeling. Their controlled and spatiotemporal delivery has therefore become a cornerstone of osteochondral tissue engineering strategies [20,37].

In osteochondral repair, MSCs are recruited to the injury site and undergo lineage-specific differentiation under the influence of signalling molecules such as BMPs, FGFs, TGF-β, and stromal cell-derived factor-1 (SDF-1) [113,123,125]. These factors may be released from the extracellular matrix, delivered via biomaterial carriers, or secreted by resident and infiltrating cells within the injury microenvironment [113,123]. Chemokines, particularly SDF-1, play a pivotal role within this recruitment program. SDF-1 acts as a potent chemoattractant for MSCs through its interaction with the CXCR4 receptor, thereby facilitating stem cell homing to osteochondral defects [113,126]. Beyond recruitment, SDF-1 contributes to angiogenesis and osteogenic differentiation, reinforcing its relevance in scaffold-based systems designed to enhance in situ regeneration [127]. However, SDF-1 is rapidly cleaved and inactivated by dipeptidyl peptidase-IV (DPP-IV), significantly reducing its bioavailability [128,129]. To address these limitations, strategies such as co-delivery with DPP-IV inhibitors, incorporation into sustained-release systems (e.g., microspheres, nanoparticles, or hydrogel-based depots), or covalent immobilization within scaffold matrices have been proposed [113,127,129]. These approaches can prolong growth factor retention, preserve bioactivity, and maintain stable chemotactic gradients, ultimately enhancing MSC recruitment and improving regenerative outcomes.

Growth factors such as platelet-derived growth factor (PDGF), epidermal growth factor (EGF), insulin-like growth factor-1 (IGF-I), hepatocyte growth factor (HGF), and FGF-2 have been shown to enhance MSC migration by activating key signalling pathways involved in chemotaxis, proliferation, and cytoskeletal remodelling [128,130]. These factors stimulate receptor-mediated downstream signalling cascades (e.g., PI3K/Akt, MAPK/ERK), thereby promoting the directional migration of MSCs toward injury sites and supporting tissue repair [131]. Combinatorial delivery of growth factors has generally been reported to further enhance MSC migration through synergistic signalling and amplification of chemotactic gradients. However, these interactions are not universally additive [128]. Certain combinations can produce antagonistic effects [132]. This highlights the importance of carefully optimizing growth factor combinations, concentrations, and temporal presentation, as complex cross-talk between signalling pathways may lead to unexpected inhibitory outcomes. Because osteochondral tissue comprises distinct yet integrated cartilage and subchondral bone compartments, bioactive cues must often be spatially organized. The spatially controlled presentation of growth factors within multilayered scaffolds has been shown to enhance phenotypic specificity, enabling cartilage formation in the superficial region and bone formation in the deeper zone [37,133]. Despite their potent bioactivity, maintaining effective local concentrations of these growth factors within biomaterial scaffolds is another translational challenge [130]. Many growth factors have short half-lives, are prone to rapid enzymatic degradation, and may diffuse rapidly from the implantation site, thereby limiting sustained chemotactic and regenerative effects.

Following recruitment, bioactive signals must direct MSC differentiation toward stable chondrogenic and osteogenic phenotypes. Growth factors represent the most extensively studied class of bioactive molecules in this regard [114,117,133]. These proteins regulate tissue development, growth, homeostasis, and repair through receptor-mediated signalling pathways. TGF-β, IGFs, FGFs, and BMPs are among the most prominent mediators of cartilage and bone formation [125]. Members of the TGF-β superfamily have been widely investigated due to their dual roles in cartilage and bone biology. TGF-β1 and TGF-β3 promote chondrogenic differentiation of MSCs and maintain cartilage-specific extracellular matrix production [134]. In parallel, BMP family members, particularly BMP-2, BMP-4, and BMP-7, demonstrate strong osteoinductive properties and have been extensively used to stimulate subchondral bone regeneration [13,135]. In addition to growth factors, small molecules provide a complementary strategy for lineage control. Kartogenin, the most extensively studied small molecule in this context, selectively induces chondrogenic differentiation of MSCs by modulating transcriptional regulators involved in cartilage gene expression [136]. Importantly, kartogenin also protects against cartilage degeneration and mitigates subchondral bone degradation in preclinical models [137]. Its chemical stability, relatively low cost, and compatibility with controlled-release systems make it attractive for incorporation into osteochondral scaffolds [138,139].

The mineral microenvironment is another important regulator of spatial differentiation within osteochondral scaffolds. In the native osteochondral unit, the transition from non-mineralized cartilage to calcified cartilage and subchondral bone is accompanied by increasing mineral content, stiffness, and osteoconductive capacity [140]. Biomaterial systems can exploit this principle by incorporating spatial gradients of mineral phases, most commonly calcium phosphate-based components such as hydroxyapatite, β-tricalcium phosphate, or other CaP-containing composites, to promote zone-specific differentiation [141,142]. Lower or absent mineral content in the cartilage compartment helps preserve a chondrogenic, non-mineralized matrix environment, whereas increasing mineralization in the deeper scaffold region supports osteogenic differentiation, matrix mineralization, and subchondral bone formation [142]. In addition to CaP phases, bioactive ions such as magnesium, silicon, strontium, and calcium may further modulate cell adhesion, osteogenesis, angiogenesis, and inflammatory responses [143,144]. Among them, divalent ions such as magnesium (Mg^2+^) have gained increasing attention due to their multifaceted roles in skeletal biology. Magnesium participates in cell adhesion, enzymatic activity, and intracellular signalling, enhances osteogenic differentiation, and modulates inflammatory responses [145,146]. In biomaterial-based systems, Mg^2+^ release can activate integrin-mediated signalling pathways, promote matrix mineralization in the subchondral region, and indirectly support chondrogenic processes, thereby influencing both bone formation and cartilage homeostasis [145,147]. Thus, mineral gradients should be viewed as instructive microenvironmental cues that help coordinate cartilage–bone compartmentalization rather than as purely structural scaffold fillers.

Successful osteochondral regeneration requires not only differentiation cues but also regulation of inflammation, angiogenesis, and extracellular matrix remodeling. Both growth factors and inorganic ions have been shown to modulate inflammatory responses and vascularization, thereby shaping the regenerative microenvironment [117,147]. Building on the use of individual growth factors, growth factor-rich biologics have emerged as an alternative strategy for delivering complex signalling environments. Platelet-rich plasma (PRP) and platelet-rich fibrin (PRF) contain diverse growth factors, including TGF-β, PDGF, vascular endothelial growth factor (VEGF), and IGF, as well as cytokines and adhesive proteins [148]. Several studies report enhanced osteochondral healing following the incorporation of platelet-derived products into scaffolds or direct application to defect sites [149,150]. However, a major limitation of these biologics is their undefined and highly variable composition, which depends on donor characteristics, preparation protocols, and activation methods [105,151]. This variability can lead to inconsistent biological responses and significant disparities in clinical outcomes, thereby limiting the reproducibility and translational reliability of these findings [152].

Collectively, these bioactive factors form a complex signalling network orchestrating osteochondral repair. Framed within a design-oriented perspective, the emphasis shifts from simple supplementation of individual molecules to the deliberate, spatiotemporal coordination of ions, chemokines, growth factors, biologics, and small molecules. Such programmable integration within advanced biomaterial platforms aims to more closely replicate the native healing cascade and achieve durable, functional osteochondral regeneration [133,139].

## 7. Spatial and Temporal Control in Osteochondral Tissue Regeneration

Native chondrogenesis unfolds through a tightly orchestrated sequence of signaling events, and leveraging these temporal dynamics offers a powerful strategy for improving cartilage regeneration. The time-dependent processes involved in native cartilage development are reviewed by Gadjanski et al. [153]. During precartilage condensation, transient activation of factors such as TGF-β, BMP-2/4/7, Wnt/β-catenin, and early-stage FGF9 promotes cell aggregation, enhances fibronectin production, and establishes the expression of adhesion molecules required for lineage commitment [154,155]. As cells transition into committed chondroprogenitors, early proliferative expansion is optimally supported by temporally restricted exposure to FGFs (particularly FGF-2 and FGF-18), which maintain chondroprogenitor identity when applied briefly but impair matrix formation when prolonged; withdrawal of FGF followed by TGF-β3 aligns with the native induction of Sox9 and the onset of early matrix deposition [156,157]. Full chondrogenic differentiation requires a subsequent shift in signaling. Sequential exposure to BMP-2 or BMP-7 and IGF-1 can enhance type II collagen and aggrecan synthesis, thereby strengthening the developing cartilage matrix and recapitulating aspects of native tissue maturation. Importantly, studies using engineered constructs indicate that timed presentation of TGF-β3 or BMPs may produce superior biochemical and mechanical outcomes compared with continuous exposure [158,159,160]. These findings support the use of staged-release biomaterials that deliver differentiation cues in a temporally controlled manner. As chondrocytes approach hypertrophy, native tissues rely on PTHrP–Ihh feedback and noncanonical Wnt signals (e.g., Wnt5b) to delay terminal differentiation; similarly, timed supplementation of PTHrP during later stages of MSC chondrogenesis suppresses hypertrophy and type X collagen expression without inhibiting cartilage matrix formation [161,162]. These temporal principles can be further reinforced through biomaterial systems designed to recapitulate developmental kinetics, such as staged-release hydrogels, MMP-sensitive matrices that degrade in synchrony with cell maturation, and stimuli-triggered factor delivery, each allowing cells to get exposed to growth factors in the same order and duration as during native cartilage formation [156]. Collectively, these insights demonstrate that effective cartilage regeneration depends not only on the choice of biochemical cues but also on recapitulating the precise timing of their presentation, mirroring the dynamic, stage-specific signaling architecture that governs embryonic cartilage development [153].

Achieving precise spatiotemporal control over tissue formation remains a central challenge for advancing effective osteochondral regeneration [133]. Unlike the repair of a single tissue type, osteochondral regeneration requires the coordinated development of two structurally and functionally distinct tissues, cartilage and subchondral bone, within a continuous and mechanically integrated unit [163,164]. A critical aspect of this challenge lies in simultaneously directing chondrogenic and osteogenic differentiation within the same regenerative system while preserving the formation of a well-defined yet functional cartilage–bone interface [164]. The need for such spatial and phenotypic control arises from the fundamental differences between cartilage and subchondral bone. Subchondral bone is a stiff, porous, and highly vascularized tissue populated by metabolically active cells that thrive in an oxygen- and nutrient-rich environment [165]. In contrast, articular cartilage is relatively soft, avascular, and characterized by low oxygen tension, limited nutrient diffusion, and a sparse cell population with low metabolic activity [166]. Due to these significant differences in mechanical properties, cellular composition, and biochemical requirements, a single-composition scaffold is rarely sufficient to support the optimal regeneration of both tissues simultaneously. In addition to supporting the formation of each tissue type, robust and durable integration at the cartilage–bone interface is essential. This interface plays a crucial role in load transfer, mechanical stability, and long-term functionality of the osteochondral unit [167]. Failure to recreate a physiologically relevant transition zone often results in delamination, poor mechanical performance, and compromised tissue durability [168].

Monolayer or monophasic scaffolds are characterized by a homogeneous composition and uniform architecture and are typically designed to replicate either the cartilage or the subchondral bone component of the osteochondral unit [169]. While such scaffolds may support the regeneration of a single tissue type, their structural and biological uniformity limits their ability to simultaneously meet the distinct, hierarchical requirements of both cartilage and bone. As a result, monophasic constructs often fail to achieve coordinated regeneration across the entire osteochondral interface. While bilayer scaffolds provided improved tissue specificity, they often faced challenges related to weak interfacial bonding and limited control over cell migration between layers, which could disrupt tissue organization and phenotypic stability [170]. Subsequently, tri-layer scaffold designs were proposed to more closely recapitulate the native osteochondral architecture [18,164]. In these constructs, an intermediate interface layer is incorporated between the cartilage and bone regions to mimic the gradual transition observed in native tissue. This interfacial layer enhances structural integrity, improves mechanical integration between layers, and enables better spatial regulation of cell behaviour by limiting uncontrolled cell migration across regions [171]. By providing distinct yet interconnected microenvironments, tri-layer systems offer improved control over lineage-specific differentiation and represent a more refined strategy for engineering functional osteochondral tissues [164].

**Figure 2:**
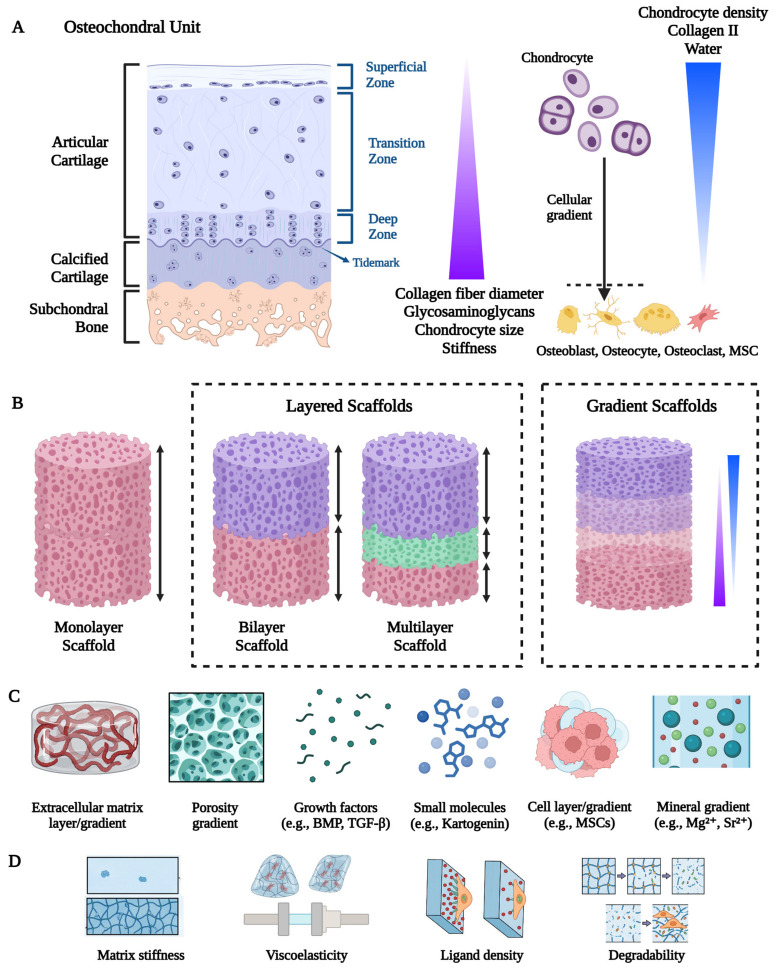
Gradients in the osteochondral unit and their translation into scaffold design strategies. (**A**) Native osteochondral organization and gradients. Schematic representation of the osteochondral unit highlighting the hierarchical architecture from articular cartilage to subchondral bone. Zonal variations in extracellular matrix composition and biophysical properties are illustrated. (**B**) Scaffold design concepts for osteochondral regeneration. Comparison of layered and gradient scaffold architectures used to recapitulate the native osteochondral interface. Monolayer scaffolds represent uniform constructs with homogeneous composition. Bilayer and multilayer scaffolds introduce discrete compartments with stepwise changes in composition, porosity, or mechanical properties, thereby mimicking the distinct regions of cartilage and bone. Gradient scaffolds exhibit continuous transitions in material composition, pore structure, and mechanical stiffness along the vertical axis, more closely simulating the gradual biochemical and biophysical gradients observed in native osteochondral tissue. (**C**) Overview of key gradient cues employed in advanced biomaterial designs, including extracellular matrix composition gradients, porosity gradients, and controlled spatial delivery of biological signals. (**D**) Additional biomaterial parameters that influence resident MSC behavior such as matrix stiffness, viscoelasticity, ligand density, and degradability. Created with BioRender.com.

To overcome these limitations, gradient scaffolds have emerged as a more biomimetic strategy for osteochondral repair [13,72,133]. These scaffolds incorporate continuous or stepwise transitions in composition, structure, and bioactivity while often being fabricated from the same base material system [172]. Gradient architectures can be generated using advanced fabrication approaches such as 3D printing, sequential or layered hydrogel deposition, and the controlled incorporation of biological cues, including growth factors or mineral phases [172,173]. By introducing gradual variations between the cartilage and bone regions, gradient scaffolds more closely replicate the native osteochondral microenvironment, facilitating spatially regulated cell differentiation and tissue formation (Figure 2) [174]. Fabrication methods must enable the scaffold to accurately match the shape and dimensions of the osteochondral defect, ensuring stable fixation and seamless integration with the surrounding host tissue [175]. In addition, the internal architecture of the scaffold should be carefully engineered to promote effective cell infiltration, nutrient diffusion, and metabolic waste removal. This requires tailoring pore size, porosity, and interconnectivity throughout the scaffold, as cartilage and subchondral bone impose fundamentally different demands on vascularization and mass transport [174,176]. Finally, because osteochondral defects frequently occur in load-bearing regions of the joint, the scaffold must possess sufficient mechanical integrity to withstand physiological stresses during the early stages of implantation [177]. Ideally, the construct should mimic the region-specific biomechanical properties of native cartilage and bone, providing temporary structural support until newly formed tissue can restore long-term mechanical function [178].

Multilayered and gradient scaffolds have consistently demonstrated superior regenerative outcomes when implanted in combination with MSCs or chondrocytes for the repair of the osteochondral unit [170,179,180]. These architectures more closely recapitulate the native spatial heterogeneity of cartilage and subchondral bone by providing zone-specific biochemical cues, mechanical properties, and structural organization [133,172]. The presence of exogenously delivered cells enables rapid matrix deposition, enhanced cell–matrix interactions, and coordinated regeneration across the cartilage–bone interface, which remain difficult to achieve using homogeneous scaffold designs [18,180]. In contrast, cell-free strategies inherently exhibit lower regenerative potential because they rely solely on host-driven repair mechanisms. Unless specifically designed to actively recruit and instruct resident progenitor or stem cells from surrounding tissues, such as subchondral bone marrow, synovium, or the cartilage periphery, acellular scaffolds often result in incomplete repair [181,182]. Even when chemotactic or bioactive cues are incorporated to enhance endogenous cell homing, achieving sufficient cell infiltration, survival, and lineage-specific differentiation remains a significant challenge. It is noteworthy that several studies have reported minimal differences in regenerative outcomes between cell-seeded and acellular scaffolds. For example, one investigation using MSC-laden scaffolds observed limited tissue specificity in the repair response [169,183], while another study incorporating autologous chondrocytes did not demonstrate enhanced glycosaminoglycan (GAG) deposition compared with an unseeded construct [184]. These findings suggest that the presence of exogenous cells does not always translate into superior osteochondral regeneration. Although cell incorporation may offer theoretical advantages by directly supplying regenerative cell populations, it also introduces substantial practical challenges. Cell-based scaffolds require extensive cell isolation, expansion, and quality control, along with increased demands for post-fabrication processing, storage, and handling. Moreover, achieving a meaningful therapeutic effect often requires delivering large numbers of cells, further increasing complexity and cost. Collectively, these factors raise concerns regarding scalability, regulatory burden, and economic feasibility, making cell-free strategies a more attractive and potentially practical alternative for osteochondral repair [185].

Beyond scaffold architecture, physicochemical cues should be treated as active design variables because they directly regulate resident MSC recruitment, phenotype, and matrix-forming capacity. Matrix stiffness is a key regulator of MSC fate: compliant 3D cartilage-like matrices that limit excessive spreading can support rounded morphology, SOX9-associated chondrogenesis, and cartilage matrix deposition, whereas stiffer substrates promote cytoskeletal tension, YAP/TAZ nuclear localization, RUNX2-associated osteogenic programs, and osteogenic differentiation [186,187,188]. In osteochondral scaffolds, this supports a zonal design in which the cartilage phase provides a compliant, chondroinductive environment, while the subchondral phase provides greater stiffness, osteoconductivity, and mechanical support [177]. Viscoelasticity is also critical, since native cartilage and many extracellular matrices exhibit stress relaxation and energy dissipation rather than purely elastic behavior. Stress-relaxing hydrogels can regulate MSC spreading, cytoskeletal organization, survival, matrix remodeling, and lineage commitment independently of initial stiffness, and appropriate relaxation behavior has been shown to enhance long-term chondrogenesis in selected hydrogel systems [189,190,191]. Therefore, hydrogel design should consider not only bulk modulus, but also relaxation time, creep behavior, and dynamic mechanical response. Ligand density and ligand identity further control MSC adhesion, spreading, migration, and differentiation. High densities of integrin-binding motifs such as RGD can increase spreading and contractility, which may favour osteogenesis or fibrotic outcomes depending on context, whereas balanced or transient adhesive ligand presentation can support migration and chondrogenesis without excessive spreading or hypertrophy [192,193]. ECM-mimetic cues such as hyaluronan, collagen type II, chondroitin sulfate, sulfated-GAG/aggrecan-mimetic motifs, and cartilage-derived peptides can help create a cartilage-like microenvironment and promote chondrogenic responses in MSCs or joint-resident progenitors [194,195,196,197,198]. Controlled degradability is equally important. Scaffolds that degrade too rapidly may lose mechanical integrity before sufficient matrix deposition, whereas overly persistent networks can restrict cell-mediated remodeling, matrix distribution, and integration. Cell-responsive degradation, particularly via MMP-sensitive linkers, enables MSCs to remodel their local matrix while maintaining structural support and has been shown to enhance chondrogenic gene expression, cartilage-specific matrix deposition, and mechanical maturation, while reducing hypertrophic changes or calcifications [199,200]. Collectively, stiffness, viscoelasticity, ligand density, degradability, and ECM-mimetic biochemical cues should be viewed as mechanistic regulators of resident MSC recruitment, lineage commitment, matrix deposition, and phenotypic stability rather than as generic scaffold properties [186,196,201].

Porosity and pore architecture play a pivotal role in directing region-specific tissue regeneration within osteochondral scaffolds [133]. In the cartilage region, smaller pore sizes have been shown to favour chondrogenesis by providing a confined microenvironment that supports chondrocyte phenotype maintenance, promotes cell condensation, and limits vascular ingrowth, all of which are essential for stable cartilage formation [202,203]. In contrast, larger pore sizes are more suitable for the subchondral bone compartment, as they enhance cell infiltration, facilitate nutrient and oxygen transport, and promote vascularization, thereby supporting osteogenesis and bone remodelling [133,202,203]. To maintain these distinct regenerative niches, an intermediate interface layer is often incorporated into osteochondral scaffolds, serving as a physical and biological barrier that separates the cartilage and bone microenvironments while enabling gradual load transfer and biochemical communication between them [133]. Beyond pore size alone, pore geometry also exerts a significant influence on cell behaviour [133]. Square pore architectures have been associated with enhanced chondrogenic differentiation, whereas rhomboidal pore geometries have been shown to preferentially promote osteogenic differentiation [204,205]. Collectively, these findings underscore the importance of spatially controlled porosity and pore shape in designing osteochondral scaffolds that can simultaneously support cartilage regeneration, subchondral bone formation, and the functional integration of the osteochondral unit [133].

A critical limitation of cell-free osteochondral scaffolds lies in the need to simultaneously satisfy competing design requirements [170]. On the one hand, the implanted construct must possess sufficient mechanical integrity to withstand the substantial compressive and shear stresses at the osteochondral interface immediately after implantation [206]. On the other hand, the scaffold architecture must remain sufficiently permissive to enable migration, proliferation, and spatial organization of resident stem cells throughout the construct [207]. Increasing scaffold stiffness to meet mechanical demands often reduces porosity and interconnectivity, thereby impairing cell infiltration and nutrient transport. Conversely, designs optimized for cellular migration frequently lack the mechanical robustness required for long-term functional integration, particularly in load-bearing joints. Therefore, the successful translation of cell-free osteochondral scaffolds will depend on advanced material strategies that decouple mechanical performance from cellular accessibility. Approaches such as dynamically stiffening hydrogels, stress-responsive materials, temporally controlled degradation profiles, and spatially graded porosity may offer solutions to this fundamental trade-off [208]. Without addressing these challenges, cell-free approaches may not match the regenerative efficacy of cell-laden, multilayered, or gradient scaffolds in restoring the structural and functional complexity of the osteochondral unit.

## 8. Niche-by-Design Framework for Endogenous MSC-Driven Osteochondral Regeneration

A niche-by-design strategy begins with clearly defining the target endogenous MSC reservoir, as different populations contribute to repair depending on defect depth, tissue disruption, and inflammatory context [209]. Superficial cartilage progenitors are most relevant in partial-thickness lesions and require low-oxygen conditions, anti-hypertrophic cues, and mechanical confinement to preserve their phenotype [6]. Synovial and synovial-fluid MSCs are highly migratory and responsive to inflammatory chemotactic signals, making them suitable targets for intra-articular biologic delivery and surface-presented chemokines [41,210]. Bone marrow-derived MSCs dominate in full-thickness defects and depend on vascular access, SDF-1 gradients, and a balanced osteogenic-chondrogenic program [15,114]. Therefore, regenerative interventions must align with the biological accessibility and functional competence of the intended niche, particularly in aging or osteoarthritic joints, where MSC responsiveness is often diminished [211].

An outcome-oriented niche should be defined not by how closely it replicates anatomy but by how effectively it orchestrates the sequence of biological events that underlie durable osteochondral repair. First, the niche must overcome the in vivo recruitment bottleneck by establishing robust chemotactic and adhesive environments for endogenous MSCs [212]. In practice, this typically involves spatiotemporally programmed chemotactic gradients (such as SDF-1/CXCR4) and complementary surface interactions (e.g., hyaluronan-CD44) to direct homing of MSCs from synovium, synovial fluid, and subchondral marrow to the defect site [95,213]. Recruitment alone, however, is insufficient for cartilage regeneration. The niche must also stabilize the chondrogenic differentiation of progenitors [14]. A frequent failure mode in cartilage repair is the formation of fibrocartilage or progression toward hypertrophic cartilage, followed by endochondral ossification [214]. Thus, the engineered environment must actively suppress fibrogenic and hypertrophic pathways while promoting stable articular chondrocyte phenotypes [215]. This may involve controlled delivery of TGF-β or BMP modulators, hypoxia-mimetic signalling, mechanical loading profiles that favour chondrogenesis, and matrix compositions enriched in glycosaminoglycans or ECM components [216,217,218]. In addition, context-appropriate signalling is necessary, underscoring the need for spatiotemporal control over bioactive signals.

Concurrently, osteochondral defects require coordinated regeneration of both hyaline cartilage and the underlying subchondral bone. These tissues differ dramatically in stiffness, vascularity, mineralization, and cellular composition [219]. Therefore, regenerative niches must either be biphasic or dynamically adaptable to provide distinct yet integrated microenvironments. Cartilage regions demand avascular, compliant, and chondroinductive conditions, whereas subchondral compartments require osteoconductive cues, higher mechanical stiffness, and permissive vascularization [220]. The success of osteochondral repair depends not only on forming each tissue independently but on establishing a mechanically and biologically coherent interface between them [206,207]. Beyond biphasic support, the niche should recreate functional gradients that drive zonal organization. Native articular cartilage exhibits depth-dependent variations in cell morphology, extracellular matrix composition, collagen fibre orientation, and mechanical properties [221]. These gradients are not merely structural features; they govern load distribution and long-term durability. Engineering spatial gradients in stiffness, growth factor concentration, oxygen tension, or mineral content can guide MSCs toward zone-specific phenotypes (superficial, middle, deep cartilage) and promote physiologically relevant tissue architecture [222].

Mechanical integration with host tissue represents another critical outcome. A regenerated construct must seamlessly integrate with surrounding cartilage and bone to prevent delamination, stress concentration, and failure under cyclic joint loading [223]. This requires materials with appropriate viscoelastic properties, interfacial adhesion, and degradation kinetics that match tissue maturation rates [224]. Mechanical cues also serve as instructive signals; thus, scaffolds should transmit physiological loading to cells in a manner that reinforces chondrogenic or osteogenic differentiation without inducing catabolic responses [206,217]. The regenerative niche must exhibit adaptive persistence, remaining functional only as long as required to guide tissue formation, and then degrading or remodelling in concert with neo-tissue maturation. Premature degradation risks loss of structural support, whereas prolonged persistence may impede matrix deposition or provoke chronic inflammation [225]. Smart biomaterials capable of cell-mediated degradation, mechanoresponsive remodelling, or environmentally triggered adaptation offer promising strategies to synchronize scaffold lifespan with regenerative progression [147,226].

A niche-by-design strategy should also distinguish several mechanistically distinct biological stages: recruitment, activation, proliferation, lineage commitment, matrix production, and functional integration. Recruitment refers to the migration or homing of endogenous progenitors into the defect and is mainly regulated by chemokines, adhesive ligands, matrix architecture, and chemotactic gradients [15,227]. Activation involves transition from a quiescent or homeostatic state toward a reparative phenotype and is influenced by injury signals, inflammatory mediators, growth factors, and matrix-derived cues [228,229]. Proliferation expands the local progenitor pool but does not, by itself, ensure regeneration [15,57]. Lineage commitment requires spatially and temporally controlled chondrogenic or osteogenic signals [230,231]. Finally, functional integration depends on matrix organization, interfacial bonding, scaffold degradation, and restoration of mechanical continuity between cartilage and subchondral bone [138,212]. Failure at any of these stages may compromise repair even if the preceding steps are successful.

A niche-by-design strategy recognizes that regenerative niches differ not only between tissues but also between patients. Age, metabolic status, inflammation, cartilage degeneration, and subchondral changes all influence MSC availability and responsiveness [232,233]. Therefore, clinically effective osteochondral niche design should integrate patient-specific factors (e.g., age, metabolic health, and inflammatory status), defect-specific factors (e.g., size, depth, location, and mechanical loading), and disease-specific factors (e.g., synovial inflammation, subchondral remodelling, and cartilage phenotype loss). This perspective implies that standardized, one-size-fits-all scaffolds may be insufficient; instead, modular or adjustable systems may be required to tailor recruitment cues and instructive signals to the individual niche profile, for example, incorporating stronger chemotactic or immunomodulatory components in aged or osteoarthritic joints [222,234].

**Figure 3:**
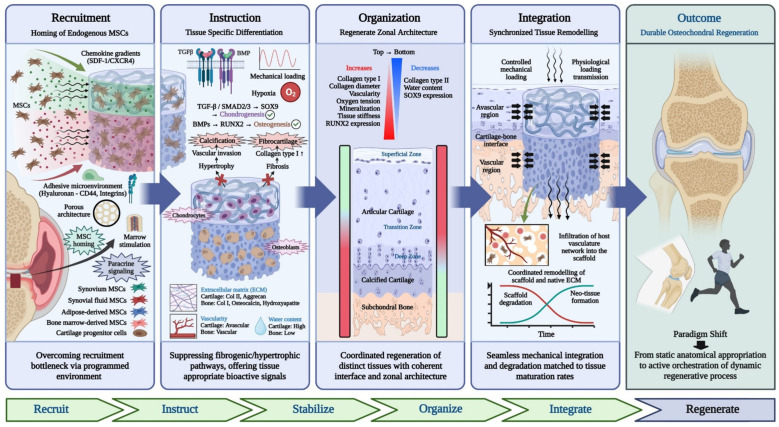
Niche-by-design framework for endogenous MSC-driven osteochondral regeneration proposing a transition from static anatomical replication to the active orchestration of biological events required for durable repair. The schematic summarizes a niche-by-design strategy in which biomaterial and microenvironmental cues first enhance recruitment and retention of endogenous progenitor cells, then direct their lineage commitment through controlled biological and mechanical signals, while spatial patterning re-establishes the hierarchical cartilage–bone architecture. Coordinated tissue formation, scaffold remodelling, and mechanical integration enable seamless interface development and functional load transfer, ultimately resulting in durable, mechanically competent osteochondral regeneration through the dynamic orchestration of host repair processes. Abbreviations: MSC: mesenchymal stem cell; ECM: extracellular matrix; TGF-β: transforming growth factor-beta; BMP: bone morphogenetic protein; SDF-1 (CXCL12): stromal cell-derived factor-1; CXCR4: CXC chemokine receptor 4; SOX9: SRY-box transcription factor 9; RUNX2: runt-related transcription factor 2; Col I/Col II: collagen type I/type II; CD44: hyaluronan receptor; O_2_: oxygen. Created with BioRender.com.

Collectively, these objectives underscore a paradigm shift in biomaterials design (Figure 3). Rather than solely striving to replicate the native extracellular matrix in static form, an outcome-oriented regenerative niche is engineered to execute a coordinated sequence of biological tasks: recruit, instruct, stabilize, organize, integrate, and ultimately relinquish control. By prioritizing key biological functions, such as cell homing, lineage stabilization, gradient establishment, and interface integration, material design shifts from merely replicating anatomy to actively coordinating dynamic regenerative processes. This systems-level approach better aligns biomaterial engineering with the complex, multiscale processes that govern successful osteochondral regeneration.

The niche-by-design framework differs from conventional biomimetic scaffold design in its primary design objective. Traditional biomimetic scaffolds often aim to reproduce selected structural or compositional features of native osteochondral tissue, such as zonal architecture, mineral gradients, porosity, or cartilage-like extracellular matrix composition. Regenerative microenvironment engineering extends this concept by incorporating biochemical or mechanical cues that support tissue formation. In contrast, the niche-by-design framework proposed here is explicitly outcome- and process-oriented: it defines the scaffold as a temporary regulatory niche that actively executes a sequence of biological functions, including resident cell recruitment, activation, retention, lineage instruction, phenotype stabilization, spatial organization, immunomodulation, interface integration, and timely remodeling. Thus, the novelty of this framework lies not in any single material component, growth factor, or scaffold architecture, but in the integration of resident MSC biology with programmable biomaterial functions. It shifts the central design question from “How closely does the scaffold resemble native tissue?” to “Which endogenous cell population should be targeted, what biological task should be induced, when should each signal be presented, and how should the material withdraw as functional tissue forms?”

## 9. Challenges and Opportunities

A critical limitation of the current literature is that many studies use endogenous cell recruitment as a surrogate for regeneration, although recruitment alone does not guarantee stable hyaline cartilage formation, prevention of hypertrophy, or durable integration with subchondral bone. The functional identity of recruited cells, their persistence within the defect, their differentiation trajectory, and their contribution to matrix organization remain incompletely resolved. Therefore, future studies should distinguish between cell homing, lineage commitment, matrix deposition, interface integration, and restoration of mechanical function as separate but interdependent outcomes.

Resident MSC populations exhibit significant heterogeneity, influenced by age and disease state. Although resident stem cells can migrate to injury sites, their recruitment is often insufficient to repair large or chronic defects. Understanding the chemotactic and matrix barriers that limit migration is one of the key aspects [235]. Another essential aspect is knowledge about precise environmental control of resident cells to avoid differentiating into fibrocartilage or hypertrophic cartilage rather than hyaline cartilage [42]. Ensuring stable and functional chondrogenesis remains a major obstacle. The osteochondral unit is a multilayered structure with complex mechanical and biochemical gradients [235]. Achieving integrated repair of cartilage, calcified cartilage, and subchondral bone is far more difficult than regenerating any single tissue. For resident stem cell strategies to succeed, they must be designed to support the entire osteochondral continuum while guiding cells toward durable, site-appropriate tissue formation [8].

More recent regenerative strategies focus on utilizing bioactive signals to actively instruct resident stem and progenitor cells to support the formation of hyaline cartilage [37]. Rather than relying solely on passive scaffolding or cell transplantation, these approaches aim to harness endogenous repair mechanisms by presenting precisely engineered biochemical and biophysical cues that regulate cell fate decisions. Biomaterials functionalized with chondrogenic bioactive motifs have emerged as promising platforms for guiding in situ cartilage regeneration [122]. Notably, fine-tuning the supramolecular dynamics of bioactive peptide amphiphiles has been shown to significantly enhance their chondrogenic bioactivity [236]. Such dynamic supramolecular systems have demonstrated an improved ability to direct resident MSCs toward a stable chondrogenic phenotype, promoting the deposition of cartilage-specific extracellular matrix components characteristic of hyaline cartilage [122,236].

Future strategies may integrate mechanical stimulation, controlled inflammation, biomaterial scaffolds, and targeted biologics to synergistically activate and guide resident stem cells. Personalized approaches that consider patient age, disease stage, and biomechanical environment are likely to become standard. Advances in single-cell RNA sequencing, proteomics, and spatial transcriptomics are revealing the diversity of resident stem cell niches at unprecedented resolution [237]. These technologies can identify subpopulations with high regenerative potential, define their signalling environments, and uncover new therapeutic targets [238].

The availability and functional competence of endogenous MSCs in the joint environment significantly impact the ability of articular cartilage to regenerate [92]. Both the quantity and biological activity of resident MSC populations gradually decrease with age and pathological conditions like osteoarthritis [239,240]. Reduced proliferative potential, decreased migratory ability, modified differentiation profiles, and decreased responsiveness to regenerative cues are some of these alterations. Furthermore, oxidative stress, matrix degradation, and chronic inflammation are common features of the aged or diseased joint microenvironment, all of which further impair MSC survival and function [239]. A major obstacle to successful articular cartilage regeneration is the interplay between intrinsic cellular deficiencies and extrinsic environmental challenges, which limits the efficacy of cartilage repair techniques that rely solely on the recruitment and activation of endogenous MSCs.

From a manufacturing perspective, acellular instructive biomaterials offer potential scalability advantages compared with cell-based therapies, which are often constrained by donor variability, expansion-induced senescence, and high production costs under GMP conditions [35,241]. Nevertheless, functionalized scaffolds incorporating growth factors, chemokines, or supramolecular peptide systems introduce their own challenges, including reproducibility of bioactive signal presentation, batch-to-batch consistency, sterilization without loss of bioactivity, and long-term storage stability [114,242]. Growth factor-based systems must also address dose control and release kinetics to avoid ectopic ossification or hypertrophic differentiation, issues previously observed with supraphysiological BMP delivery in orthopaedic applications [242]. Thus, translation will depend on the development of biomaterials with predictable pharmacokinetic and pharmacodynamic profiles that meet regulatory standards for combination products.

Regulatory pathways also shape translational feasibility [33,34]. Endogenous MSC-activating scaffolds without viable cells may be regulated as medical devices or combination products, potentially offering a more streamlined approval process compared with advanced therapy medicinal products (ATMPs) involving manipulated cells. However, once bioactive molecules with pharmacological action are incorporated, regulatory classification may shift, requiring extensive safety, toxicology, and biodistribution data [243]. Early dialogue with regulatory agencies is therefore critical to define classification, preclinical study requirements, and clinical trial design. Lessons from previously approved cartilage repair products underscore the importance of long-term post-market surveillance to evaluate durability and delayed adverse events.

Although resident MSC-targeting strategies offer an attractive route toward cell-free and in situ osteochondral regeneration, several unresolved controversies limit their current translational maturity. The identity and functional equivalence of joint-resident MSC populations remain incompletely defined. Surface marker panels such as CD73, CD90, CD105, CD44, CD146, CD271, and STRO-1 are useful for phenotypic enrichment, but they do not necessarily identify a uniform regenerative population across cartilage, synovium, synovial fluid, periosteum, infrapatellar fat pad, and bone marrow [54,244]. Consequently, studies that report resident MSC recruitment may involve biologically distinct progenitor subsets with different migratory, immunomodulatory, chondrogenic, osteogenic, or hypertrophic tendencies. Similarly, the inflammatory joint environment may simultaneously mobilize progenitor cells and impair their regenerative function, creating a paradox in which the same cues that enhance recruitment may compromise long-term phenotypic stability [245,246].

In addition, the therapeutic potential of resident MSC-targeting strategies must be interpreted cautiously in advanced osteoarthritis. In early or focal osteochondral defects, endogenous progenitor populations may retain sufficient migratory and differentiation capacity to support repair when appropriately stimulated [247,248]. In contrast, advanced osteoarthritis is characterized by chronic synovial inflammation, oxidative stress, cellular senescence, extracellular matrix degradation, altered subchondral bone remodeling, vascular invasion, and changes in joint biomechanics [249,250]. These features can impair MSC proliferation, migration, chondrogenic differentiation, and responsiveness to regenerative cues.

A critical unresolved question in the field is whether resident MSC-targeting strategies can consistently outperform cell-based therapies. Cell-free scaffolds and chemotactic biomaterials avoid many manufacturing and regulatory burdens associated with expanded MSC products, but their efficacy depends on the availability, responsiveness, and spatial organization of host progenitors [247,248]. These requirements may not be met in aged, osteoarthritic, or highly inflamed joints. Despite encouraging preclinical findings, most resident MSC-targeting strategies remain supported primarily by in vitro and small-animal studies. Although large-animal models and early clinical investigations are emerging, current evidence remains insufficient to establish their long-term durability, functional integration, ability to prevent fibrocartilage formation, or capacity to restore the structural and mechanical properties of native osteochondral tissue [11,12]. Future studies should therefore move beyond conventional histological repair scores and adopt standardized, multidimensional evaluation frameworks that assess endogenous cell recruitment, lineage commitment, extracellular matrix composition and organization, tissue interface integration, biomechanical performance, functional outcomes, and long-term safety. Such a comprehensive evaluation will be critical for accurately determining the translational potential of resident MSC-targeting therapies.

Emerging technologies may accelerate the development of personalized niche-by-design strategies. Single-cell RNA sequencing and single-cell multi-omics can resolve the heterogeneity of resident MSC populations and identify progenitor subsets with high regenerative or immunomodulatory potential [249,251]. Spatial transcriptomics could map how these populations interact with inflammatory, vascular, and matrix niches across the osteochondral unit. In addition, AI-assisted biomaterial design and computational screening may help predict combinations of stiffness, ligand density, degradability, growth factor release, and scaffold architecture that best match patient- and defect-specific requirements [252]. Together, these approaches may enable a transition from empirical scaffold development toward data-guided, patient-tailored regenerative niche engineering.

## 10. Clinical Evidence

Translation of resident MSC-targeting strategies requires a clearer understanding of which endogenous cell populations are engaged by currently available interventions. Marrow stimulation procedures primarily target bone marrow-derived MSCs by breaching the subchondral plate, whereas scaffold-augmented microfracture attempts to stabilize the marrow clot and improve chondrogenic instruction [100,253,254]. Intra-articular biologics and chemokine-releasing systems may target synovial, synovial-fluid, IFP-derived, and marrow-derived progenitors, but the relative contribution of each population remains difficult to quantify in patients [54,60]. Similarly, biomimetic gradient scaffolds may recruit cells from multiple adjacent compartments, including subchondral marrow, cartilage margins, synovium, and periosteum, depending on defect geometry and implantation site [255]. A major unresolved translational question is the threshold of endogenous cell recruitment required for meaningful repair. Current studies often report increased cell migration or scaffold infiltration, but few define the minimum number, phenotype, spatial distribution, or persistence of recruited cells required to generate mechanically competent hyaline-like cartilage and integrated subchondral bone [256]. This issue is particularly important in aged or osteoarthritic joints, where proliferative capacity, migration, and differentiation potential may be reduced [232]. Patient-related factors such as age, metabolic status, inflammatory burden, synovitis, subchondral bone remodeling, and lesion chronicity should therefore be incorporated into future trial design and biomaterial selection [249,250].

A preliminary search of the ClinicalTrials.gov database using the keywords “osteochondral” and “stem cells” identified only a few studies, highlighting the limited number of clinical investigations specifically targeting osteochondral defects or lesions. We then refined the search to focus exclusively on osteochondral conditions and manually screened the resulting list to identify studies leveraging endogenous or resident stem cell-based approaches. The key clinical studies identified through this process are summarized in Supplementary Table S1. Overall, the current landscape demonstrates that most advancements remain at the in vitro or preclinical stage, with only a handful of early-phase clinical studies underway. This underscores the need for well-designed clinical trials to translate emerging biological repair strategies, particularly those that rely on endogenous stem cell activation, into clinically validated therapies for osteochondral regeneration.

**Figure 4:**
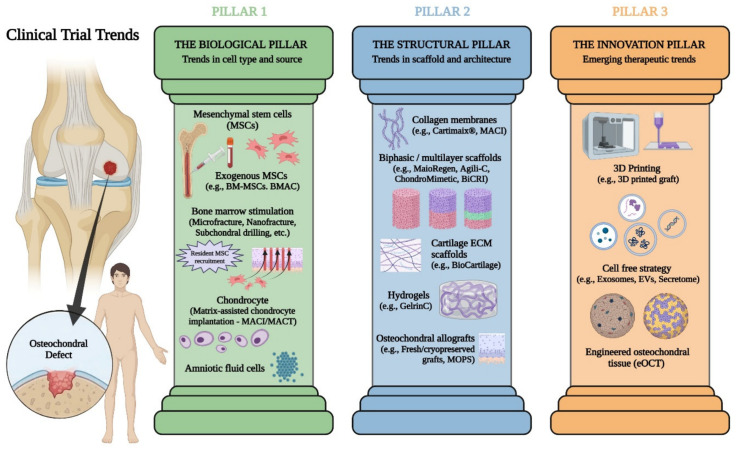
Therapeutic landscape of osteochondral clinical research. Interventions are categorized into three strategic pillars: (1) Biological, focusing on cell delivery (MSCs, BMAC) and resident cell recruitment (nanofracture); (2) Structural, emphasizing biomimetic scaffolds (biphasic/triphasic) and allografts; and (3) Innovation, highlighting 3D printing, cell-free secretome, and in vitro engineered tissues. Created with BioRender.com.

The therapeutic landscape of osteochondral clinical research reflects a convergence of regenerative biology, biomaterials engineering, and emerging technologies. Current clinical trials can broadly be organized into three strategic pillars (Figure 4). Biological approaches focus on enhancing the regenerative potential of osteochondral defects through cell-based therapies, including MSCs and bone marrow aspirate concentrate (BMAC), as well as resident cell recruitment strategies, such as microfracture or nanofracture, that stimulate endogenous progenitor cells in the subchondral bone. Structural strategies aim to restore the architecture of the osteochondral unit using biomimetic scaffolds, including biphasic and triphasic constructs that replicate cartilage–bone gradients, as well as osteochondral allografts that provide immediate structural and biological support. In parallel, an innovation-driven pillar is emerging, incorporating advanced technologies such as 3D-printed grafts, cell-free secretome therapies, and in vitro-engineered osteochondral tissues, aiming to improve defect-specific repair and enhance regenerative outcomes. Collectively, these strategies illustrate a shift toward integrated approaches that combine biological stimulation with structural restoration to achieve more durable osteochondral regeneration.

Current clinical evidence for resident MSC-targeted osteochondral regeneration remains limited compared with the extensive preclinical literature. Most clinically used strategies that engage endogenous progenitor cells are marrow stimulation-based procedures, including microfracture, drilling, nanofracture, and related scaffold-augmented techniques. These approaches primarily target bone marrow-derived MSCs by allowing marrow elements to enter the defect site. Although they can improve symptoms and promote defect filling, the repair tissue is frequently fibrocartilaginous and may demonstrate limited long-term durability, particularly in larger lesions or high-demand patients [11,12]. Clinical strategies using biomaterial augmentation attempt to improve upon marrow stimulation by stabilizing the clot, enhancing cell retention, and providing chondrogenic matrix cues. Other trials investigate BMAC, MSC-seeded scaffolds, osteochondral grafts, 3D-printed constructs, and cell-free secretome or extracellular vesicle-based approaches. However, direct clinical evidence for the precise targeting of synovial MSCs, synovial fluid MSCs, IFP-derived MSCs, cartilage-resident MSCs, or periosteal MSCs remains comparatively sparse. In most clinical studies, the exact endogenous cell source contributing to repair is inferred rather than directly demonstrated. The main translational barriers include limited long-term evidence of hyaline cartilage regeneration, variability in patient selection, inconsistent lesion characteristics, lack of standardized outcome measures, unclear potency metrics for endogenous cell recruitment, and difficulty distinguishing biological repair from symptomatic improvement. Future trials should incorporate imaging, functional outcomes, biomarkers of inflammation and matrix turnover, and, where feasible, histological or compositional assessment of repair tissue quality.

The clinical trials listed in Supplementary Table S1 highlight the broad and evolving landscape of regenerative strategies being explored to treat osteochondral defects, particularly in the knee joint. Many trials specifically investigate the use of stem or progenitor cell sources, either by directly implanting exogenous cells or by stimulating resident bone marrow stem cells. Together, these studies illustrate the growing emphasis on biologically driven repair strategies aimed at restoring both cartilage and subchondral bone architecture. A significant subset of trials focuses on MSC-based therapies, highlighting the promise of these cells in cartilage and osteochondral regeneration. Approaches include the implantation of autologous bone marrow–derived MSCs, either culture-expanded or freshly isolated, and the use of BMAC to deliver a heterogeneous population of progenitor cells to the defect site. Some studies combine MSCs with biomaterial scaffolds or osteochondral grafts to enhance cell retention and tissue integration, while others investigate engineered osteochondral tissues derived from patient-derived MSCs. In addition to direct cell implantation, emerging strategies explore the paracrine effects of stem cells, such as the therapeutic use of the secretome of adipose-derived MSCs or extracellular vesicles, reflecting a growing interest in cell-free regenerative therapies. Another major group of clinical trials investigates techniques that recruit endogenous stem cells from the bone marrow to facilitate repair. Procedures such as microfracture, nanofracture, and matrix-assisted autologous matrix-induced chondrogenesis are designed to stimulate bone marrow–derived progenitor cell migration into the defect area. These approaches are often augmented with biomaterials, such as collagen membranes, cartilage matrices, or hydrogels, which stabilize the blood clot, support cell attachment, and promote chondrogenic differentiation. Such strategies aim to harness the body’s intrinsic repair mechanisms while improving the quality and durability of regenerated tissue.

Surgical handling and fixation represent additional determinants of clinical success. Osteochondral lesions frequently occur in load-bearing zones that require immediate mechanical stability. Biomaterials designed to recruit resident MSCs must therefore possess adequate compressive strength, integrate with subchondral bone, and resist delamination under shear stress [222,235]. Techniques such as press-fit implantation, bioresorbable pins, fibrin adhesives, or osteochondral plug–like constructs may be required depending on defect size and location [257]. Importantly, surgical complexity directly affects adoption; minimally invasive, single-stage procedures are more likely to gain widespread clinical acceptance compared with multi-stage or technically demanding interventions [258].

## 11. Conclusions

Resident MSCs represent one of the most promising avenues for functional osteochondral regeneration. By leveraging populations already conditioned by the joint environment, endogenous repair strategies aim to overcome the variability and limitations of exogenous cell therapies. Significant progress has been made in identifying resident progenitor niches, understanding key regulatory signals, and developing technologies that activate or guide these cells. However, challenges related to heterogeneity, migration, control of differentiation, and integration across the osteochondral interface remain formidable. The path forward lies in more precise mapping of stem cell niches, improved understanding of mechanobiology, and the development of biomaterials and biologics that can recreate native regenerative cues. As the field moves toward minimally manipulated, patient-specific therapies, resident MSCs may become central to the next generation of osteochondral repair strategies. The key question is how we can unlock and orchestrate the regenerative capacity already present within the joint.

Effective osteochondral regeneration requires more than the restoration of cartilage or bone in isolation. It demands precise spatial and phenotypic control to recreate two fundamentally distinct tissues within a continuous, mechanically integrated unit. The evidence discussed highlights that homogeneous scaffold designs are inherently limited in their ability to address the divergent biological, mechanical, and metabolic requirements of articular cartilage and subchondral bone. As a result, monophasic constructs frequently fail to achieve durable integration or long-term functional repair. Gradient and multilayered scaffold architectures represent a significant advancement toward biomimetic osteochondral regeneration. By introducing spatial variations in composition, porosity, stiffness, and bioactivity, these designs more closely replicate the native osteochondral organization and may promote region-specific chondrogenic and osteogenic differentiation. Future progress in osteochondral tissue engineering will depend on advanced material strategies that decouple mechanical performance from porosity and cell migration, while preserving spatially defined biological cues. Integrating graded architectures with dynamic, responsive materials offers a promising pathway toward achieving durable, functional regeneration of the osteochondral unit. Approaches such as dynamically stiffening hydrogels, stress-responsive materials, temporally controlled degradation, and spatially graded porosity may offer viable solutions.

The niche-by-design framework represents the central conceptual advance proposed in this review. Rather than treating osteochondral scaffolds as passive templates or as carriers for exogenous cells, this framework positions biomaterials as programmable regulators of the endogenous repair niche. In this view, an effective scaffold should sequentially recruit resident MSCs, retain them within the defect, instruct their region-specific differentiation, stabilize a non-hypertrophic chondrogenic phenotype in the cartilage compartment, support osteogenesis in the subchondral compartment, modulate inflammation, and ultimately remodel in synchrony with new tissue formation. Translationally, this framework supports a shift from universal scaffold designs to modular, patient-responsive regenerative systems. Defect depth, lesion size, joint loading environment, age, inflammatory status, osteoarthritis severity, and subchondral bone remodeling should inform the selection of chemotactic, immunomodulatory, chondrogenic, osteogenic, and mechanical cues incorporated into the biomaterial. For early or superficial lesions, designs targeting synovial, synovial fluid, or superficial cartilage progenitors may be prioritized, whereas full-thickness osteochondral defects may require stronger engagement of bone marrow-derived MSCs and spatially organized cartilage–bone instructive gradients. Future translation will require resident MSC-targeting strategies to meet several benchmarks: reproducible manufacturing, stable presentation of bioactive cues, compatibility with single-stage surgical workflows, clear regulatory classification, and clinically meaningful evidence of durable hyaline cartilage formation and osteochondral integration. If these challenges can be addressed, niche-by-design biomaterials may provide a scalable route toward endogenous, cell-free, and patient-specific osteochondral regeneration.

## Figures and Tables

**Table 1:** Comparative appraisal of joint-resident MSC sources for osteochondral regeneration.

MSC Source	Proliferation	Chondrogenic Potential	Accessibility	Clinical Relevance	Ref.
Cartilage-resident progenitors	Low in situ abundance but moderate-to-high proliferative/clonogenic capacity after isolation and expansion.	Highly chondrogenic, capable of generating hyaline-like cartilage with relatively low hypertrophic tendency; particularly relevant for superficial cartilage maintenance and repair.	Poor; embedded within dense cartilage ECM and typically require tissue harvest.	Particularly relevant for early/superficial lesions, cartilage homeostasis, and integration with native cartilage.	[56,57,58]
Synovial membrane MSCs	High proliferative capacity and colony-forming ability.	High chondrogenic potential; frequently regarded as one of the most chondrogenic joint-resident MSC populations.	Moderate; accessible arthroscopically or through synovial biopsy.	Highly relevant for cartilage repair, meniscal regeneration, and one-stage cell-based procedures.	[39,55]
Synovial fluid MSCs	Moderate-to-high proliferation, with cell numbers increasing after joint injury, inflammation, or synovitis.	Moderate-to-high chondrogenic capacity; responsive to chondrogenic stimulation, although not consistently superior to BM-MSCs.	High; can be harvested minimally invasively from joint aspirates.	Attractive for endogenous cell mobilization, intra-articular biologic therapies, and minimally invasive regenerative strategies.	[52,53,54]
Bone marrow-derived MSCs	Moderate proliferation with robust ex vivo expansion capacity.	Strong chondrogenic and osteogenic differentiation potential, making them central to osteochondral repair.	High; available through marrow stimulation techniques, bone marrow aspiration concentrate (BMAC), or iliac crest harvest.	Most clinically established MSC source; widely used in microfracture augmentation, drilling, nanofracture, and BMAC procedures.	[54,62,63]
Periosteum-derived progenitors	High proliferative activity, particularly following injury-induced activation.	Strong chondrogenic and osteogenic capacity, with an important role in endochondral ossification and osteochondral interface regeneration.	Moderate; harvesting is more invasive than synovial or synovial-fluid sources.	Particularly relevant for osteochondral interface reconstruction, fracture healing, and endochondral repair strategies.	[64,65,66]
IFP/adipose-derived MSCs	High proliferation and good expansion potential, including cells isolated from osteoarthritic joints.	Moderate-to-high chondrogenic potential; often superior to subcutaneous adipose MSCs and capable of producing robust cartilaginous matrix under appropriate stimulation.	High; readily obtained during knee arthroscopy or open knee surgery.	Clinically attractive resident adipose source with chondrogenic and immunomodulatory properties.	[59,60,61]

**Table 2:** Characteristics of different resident mesenchymal stem cells and potential targeting strategies.

Resident Stem Cell Source	Surface Markers	Targeting Strategies
Articular cartilage	Core MSC identity: CD73^+^, CD90^+^, CD105^+^, STRO-1^+^, CD146^+^, CD166^+^ Adhesion markers: CD29^+^, CD44^+^, CD166^+^, CD146^+^, CD90^+^ Homing/Niche signaling: Notch1^+^, CD44^+^, CD29^+^, CD146^+^ Immunophenotypic exclusion: CD11b^−^, CD14^−^, CD19^−^, CD34^−^, CD45^−^, CD79a^−^, HLA-DR^−^	Multiple soluble factors have been identified as key regulators of the migration and activation of resident cartilage progenitor cells. These include high mobility group box 1 (HMGB1), which stimulates progenitor cell mobilization following tissue injury, as well as insulin-like growth factor 1 (IGF-1) and platelet-derived growth factor (PDGF) [6]. Intermittent hydrostatic pressure (IHP) has been shown to significantly enhance the chondrogenic differentiation of cartilage progenitor cells when cultured within alginate bead systems, highlighting the important role of physiologically relevant mechanical stimulation in promoting cartilage-specific matrix formation and phenotype stabilization [109]. Platelet lysates recruit cartilage progenitor cells that can contribute to tissue repair [110].
Bone marrow	Core MSC identity: CD73^+^, CD90^+^, CD105^+^, CD271^+^, STRO-1^+^, CD146^+^ Adhesion markers: CD29^+^, CD44^+^, CD166^+^, CD146^+^, CD147^+^ Homing/Migration: CD271^+^, CD44^+^, CD29^+^, CD146^+^, CD147^+^ Immunophenotypic exclusion: CD11b^−^, CD14^−^, CD19^−^, CD34^−^, CD45^−^, CD79a^−^, HLA-DR^−^	The chemokine stromal cell-derived factor 1 (SDF-1) has been widely investigated as a strategy to recruit bone marrow–derived mesenchymal stem cells (BM-MSCs) to sites of cartilage injury through activation of the SDF-1/CXCR4 signalling axis. Incorporation of SDF-1 into biomaterial scaffolds enhances endogenous cell homing to the defect region, thereby promoting cartilage repair, evidenced by increased production of type II collagen and glycosaminoglycans [15]. Platelet-rich plasma can be applied at the site of injury to increase the local availability of BM-MSCs by enhancing their recruitment and proliferative activity. Through the release of a concentrated pool of growth factors and chemokines, platelet-rich plasma creates a pro-regenerative microenvironment that supports endogenous cell homing and activation, thereby contributing to improved repair of articular cartilage [111]. BM-MSCs contained within autologous bone marrow aspirate concentrate can be delivered in combination with a hyaluronan-based scaffold using a single-step surgical procedure, enabling efficient cell delivery and scaffold implantation within the same operative setting [112]. A composite hydrogel scaffold has been developed by integrating an oriented acellular cartilage matrix with a bone marrow homing peptide-functionalized self-assembling peptide to enhance endogenous MSC homing and promote chondrogenic differentiation. This biomimetic strategy significantly enhances the therapeutic efficacy of cartilage repair by facilitating targeted cell recruitment, promoting lineage-specific differentiation, and promoting functional regeneration of chondral defects [14]. LIPUS-assisted (low-intensity pulsed ultrasound) SDF-1/BMP-2 nanoparticle system has been shown to effectively recruit BM-MSCs, thereby enhancing endogenous cell homing to the target site [113]. The combined delivery of platelet-derived growth factor BB (PDGF-BB) and transforming growth factor β1 (TGF-β1) significantly stimulated the migration of bone marrow–derived progenitor cells [114]. Fibroblast growth factor (FGF2) promotes the migration of BM-MSCs [115].
Periosteum	Core MSC identity: CD73^+^, CD90^+^, CD105^+^, STRO-1^+^ Adhesion markers: CD29^+^, CD44^+^, CD166^+^ Homing/Migration: CD44^+^, CD29^+^ Immunophenotypic exclusion: CD14^−^, CD33^−^, CD34^−^, CD45^−^, CD133^−^, HLA-DR^−^	Bone morphogenetic protein 2 (BMP-2)–loaded scaffolds can be employed to induce endochondral ossification by promoting the recruitment and differentiation of periosteum-derived MSCs. In addition, incorporating chondroitin sulphate as a bioactive enhancer can further increase the abundance of recruited cells and support their osteochondral differentiation, thereby enhancing both the recruitment efficiency and regenerative capacity of the scaffold [116].
Synovial membrane	Core MSC identity: CD90^+^, CD105^+^, CD271^+^ Adhesion markers: CD44^+^, CD147^+^ Homing/Migration: CD271^+^, CD44^+^, CD147^+^ Immunophenotypic exclusion: CD31^−^, CD34^−^, CD45^−^, CD177^−^, HLA-DR^−^	A three-phase polydopamine modified osteochondral bionic scaffold was engineered by integrating hydroxyapatite and silk fibroin, with controlled release of platelet-derived growth factor. This biomimetic construct was specifically designed to promote efficient recruitment of synovial membrane–derived mesenchymal stem cells, thereby enhancing endogenous cell homing and facilitating coordinated osteochondral repair [117]. The efficacy of a scaffold-free, tissue-engineered construct based on autologous synovial mesenchymal stem cells has been demonstrated for regenerative cartilage repair, with favourable clinical outcomes and supportive magnetic resonance imaging findings at midterm follow-up [118]. The combined delivery of TGF-β3 and SDF-1β exerted a strong chemotactic effect on synovium-derived stem cells and markedly enhanced the expression of aggrecan and type II collagen genes [41].
Synovial fluid	Core MSC identity: CD90^+^, CD105^+^, CD271^+^, UDPGD^+^ Adhesion markers: CD44^+^ Homing/Migration: CD271^+^, CD44^+^ Immunomodulatory/Activation: CD40^+^ Immunophenotypic Exclusion: CD11b^−^, CD19^−^, CD34^−^, CD45^−^, HLA-DR^−^	Non-culture expanded synovial-mobilized mesenchymal stem cells (Sm-MSCs) and synovial fluid microfragments can be enriched using a purpose-built stem cell mobilization device (STEM device). Non-culture expanded synovial fluid-derived MSCs and synovial fluid microfragments have been shown to possess intrinsic chondrogenic potential, enabling cartilage formation [52]. Platelet lysates enhanced the adhesion, proliferation, migration, and chondrogenic differentiation capacity of synovial fluid-derived MSC [119].
Infrapatellar fat pad	Core MSC identity: CD90^+^, CD105^+^, CD13^+^ Adhesion markers: CD29^+^, CD44^+^, CD13^+^ Homing/Migration: CD44^+^, CD29^+^ Immunophenotypic exclusion: CD34^−^, CD56^−^, CD271^−^, STRO-1^−^	MSCs derived from the infrapatellar fat pad (IFP) can undergo robust chondrogenic differentiation within 3D printed chitosan scaffolds when stimulated with TGF-β3 and BMP6 [120]. Cartilage-derived morphogenetic protein-1 (CDMP-1) and osteogenic protein-1 (OP-1) may modulate the abundance, differentiation capacity, and functional properties of IFP-MSCs, thereby influencing their contribution to cartilage regeneration [121].

## Data Availability

No new data were created or analyzed in this study. Data sharing is not applicable to this article.

## Supplementary Materials

The following supporting information can be downloaded at https://www.mdpi.com/article/10.3390/cells15141290/s1, Table S1: Clinical trials registered in the ClinicalTrials.gov database highlighting different therapeutic strategies that leverage exogenous or resident stem cell sources for the treatment of osteochondral defects.

## Abbreviations

The following abbreviations are used in this manuscript:

ATMP	Advanced therapy medicinal product
BMAC	Bone marrow aspirate concentrate
BM-MSCs	Bone marrow-derived mesenchymal stem cells
BMP(s)	Bone morphogenetic protein(s)
CDMP-1	Cartilage-derived morphogenetic protein-1
CXCR4	CXC chemokine receptor 4
DPP-IV	Dipeptidyl peptidase IV
ECM	Extracellular matrix
EGF	Epidermal growth factor
FGF(s)	Fibroblast growth factor(s)
FGF-2/FGF-18	Fibroblast growth factor 2/18
GAG	Glycosaminoglycan
GMP	Good manufacturing practice
HGF	Hepatocyte growth factor
HLA-DR	Human leukocyte antigen—DR isotype
HMGB1	High mobility group box 1
IFP	Infrapatellar fat pad
IGF/IGF-1	Insulin-like growth factor/Insulin-like growth factor 1
IHP	Intermittent hydrostatic pressure
LIPUS	Low-intensity pulsed ultrasound
MSC(s)	Mesenchymal stem cell(s)
OA	Osteoarthritis
OP-1	Osteogenic protein-1
