# Supplementary material for: Design Strategies to Target Joint Resident Mesenchymal Stem Cells for Osteochondral Regeneration
Source: Cells. 2026 Jul 18;15(14):1290. doi: 10.3390/cells15141290 (PMC13406706; doi:10.3390/cells15141290)
Supplement: Supplementary file 1 [file cells-15-01290-s001.zip › cells-4414133-supplementary.pdf]

Supplementary Table S1: Clinical trials registered in the ClinicalTrials.gov database highlighting different therapeutic strategies that leverage exogenous or resident stem cell sources for the treatment of osteochondral defects.

Trial ID	Title	Intervention	Status
"Osteochondral" AND "Stem cell"			
NCT06078072	Biomaterials and Mesenchymal Stem/Stromal Cells in the Treatment of Knee Articular Surface Lesions	Combined single-step procedure of treating knee articular surface lesions with biomaterials (scaffolds) and mesenchymal stem cells (filtered bone marrow aspirate concentrate)	Completed
NCT00891501	The Use of Autologous Bone Marrow Mesenchymal Stem Cells in the Treatment of Articular Cartilage Defects	Implantation of autologous, culture-expanded, mesenchymal stem cells obtained from the bone marrow of patients	Recruiting
NCT04223622	Effects of ASC Secretome on Human Osteochondral Explants (ASC-OA)	Osteochondral explants obtained from arthroplasty patients are induced to develop an osteoarthritis (OA)-like phenotype. They are then treated with adipose-derived stromal cell (ASC) secretome either as complete conditioned medium or as isolated extracellular vesicles, to evaluate its therapeutic potential.	Completed
NCT03625180	An Observational, Prospective Study of Patients with Chondral and/or Osteochondral Defects of the Knee Treated with NAMIC (KNAMIC)	NAMIC (Nanofractured Autologous Matrix-Induced Chondrogenesis) is a cartilage repair technique in which the damaged cartilage is first carefully removed while preserving the surrounding healthy cartilage. The calcified cartilage layer is also removed. The underlying bone is then stimulated using a minimally invasive nanofracture technique (NanoFX, Arthrosurface, USA) to release bone marrow cells. Finally, the defect is covered with a type I/III	Recruiting

		collagen–elastin membrane (Cartimaix, Matricel, Germany) to protect the area, stabilize the blood clot, and support the attachment and growth of stem cells migrating from the bone marrow.	
NCT04739930	Autologous Bone Marrow Concentrate in Knee Osteochondral Allograft Transplantation	The intervention involves processing bone marrow aspirate to obtain a concentrate, soaking the osteochondral allograft plug in the concentrate prior to implantation, and applying the remaining concentrate to the defect site before placing the plug.	Completed
NCT06400862	Treating Patients with Traumatic Chondral Lesions with Autologous Bone Marrow Cells Derived Engineered Tissues - Engineered Osteochondral Tissue	The intervention involves harvesting autologous bone marrow to isolate mesenchymal stem/stromal cells (MSCs) for the manufacture of engineered osteochondral tissue (eOCT). Approximately 13 weeks later, the cartilage lesion is treated by arthroscopic implantation of the autologous eOCT.	Recruiting
NCT05651997	Study Comparing Two Methods for the Treatment of Large Chondral and Osteochondral Defects of the Knee	Matrix-Assisted Autologous Chondrocyte Transplantation (MACT) involves harvesting autologous cartilage cells, expanding them in a laboratory, seeding them onto a type I/III collagen membrane, and implanting the cell-seeded matrix into the cartilage defect to promote repair. Augmented Microfracture Technique (AMT/AMIC) combines microfracture of the subchondral bone with the application of a type I/III collagen membrane to cover the defect, aiming to stabilize the blood clot, enhance mesenchymal stem cell retention, and support cartilage regeneration.	Not yet recruiting

NCT01159899	Transplantation of Bone Marrow Stem Cells Stimulated by Protein Scaffolds to Heal Defects Articular Cartilage of the Knee	This pilot study investigates the arthroscopic transplantation of freshly isolated, non-cultured autologous bone marrow mesenchymal stem cells (MSCs) mixed with a protein scaffold and collagen–hydroxyapatite matrix in patients.	Recruiting
"Osteochondral"			
NCT06216756	Evaluating Cryopreserved Osteochondral Allograft Cores for the Treatment of Osteochondral Lesions in the Knee	This intervention involves transplantation of one or more cryopreserved osteochondral allograft cores to repair a defect in the femoral condyle.	Recruiting
NCT04236492	Study Protocol to Evaluate Clinical and Imaging Results of Knee Fresh Osteochondral Allografts	This intervention involves transplantation of a fresh osteochondral allograft into the knee to repair the osteochondral defect.	Recruiting
NCT03777735	Human Bone Graft for Fixation of Osteochondral Defects in the Knee Joint	This observational study evaluates the use of human bone screw grafts (Shark Screw®) for the surgical treatment of osteochondral defects.	Recruiting
NCT03696394	A Study to Evaluate the Efficacy of BioCartilage® Micronized Cartilage Matrix in Microfracture Treatment of Osteochondral Defects	This intervention consists of microfracture of the cartilage defect followed by application of BioCartilage® micronized cartilage matrix, a scaffold containing type II collagen and cartilage matrix components, to support cartilage repair.	Recruiting
NCT01209390	A Prospective, Post-marketing Registry on the Use of ChondroMimetic for the Repair of Osteochondral Defects	This intervention involves implanting the ChondroMimetic osteochondral scaffold into the defect site. The biphasic plug consists of a chondral layer made of collagen and glycosaminoglycans (GAG) and a bone layer composed of collagen, GAG, and calcium phosphate, designed to support bone and cartilage tissue ingrowth.	Terminated

NCT03385642	Follow-Up Study Evaluating the Long-Term Outcome of ChondroMimetic in the Treatment of Osteochondral Defects in the Knee	This intervention involves implantation of ChondroMimetic, a single-use biphasic osteochondral scaffold, into the knee to support repair of cartilage defects.	Completed
NCT03036878	ReNu™ Marrow Stimulation Augmentation	This intervention involves the use of ReNu™ allograft as an adjunct to standard marrow stimulation for the treatment of osteochondral defects. ReNu™ is an allograft tissue derived from particulated amniotic membrane and cells obtained from amniotic fluid.	Terminated
NCT06895889	EB-OC for the Treatment of Focal Chondral/Osteochondral Defects in the Knee	This intervention involves implantation of EB-OC, a tissue-engineered osteochondral graft composed of a living engineered cartilage layer attached to a bone scaffold, for the repair of full-thickness chondral or osteochondral defects of the knee, and comparison with abrasion chondroplasty.	Not yet recruiting
NCT00945399	Comparison of Microfracture Treatment and CARTIPATCH® Chondrocyte Graft Treatment in Femoral Condyle Lesions	This intervention involves harvesting autologous cartilage, isolating and expanding chondrocytes in culture, seeding them onto a biomaterial scaffold (CARTIPATCH®), and surgically implanting the construct into the cartilage defect. Outcomes are compared with a microfracture treatment group.	Terminated
NCT00560664	Comparison of Autologous Chondrocyte Implantation Versus Mosaicoplasty: a Randomized Trial (Cartipatch)	This intervention involves harvesting autologous cartilage, isolating and expanding chondrocytes in culture, embedding the cells in an agarose matrix, and surgically implanting the construct into the defect at a later procedure. Outcomes are compared with a mosaicoplasty treatment group.	Completed
NCT01282034	Study for the Treatment of Knee Chondral and Osteochondral Lesions	This intervention involves implantation of MaioRegen®, a multi-layered bioceramic scaffold composed of	Completed

		deantigenated type I equine collagen and magnesium-enriched hydroxyapatite, designed to replicate the full osteochondral structure. Its performance is compared with standard surgical techniques, such as microfracture and subchondral drilling, for the treatment of chondral or osteochondral lesions.	
NCT02308358	Long-Term Outcomes of Osteochondral Allografts for Osteochondral Defects of the Knee	Subjects with femoral condyle osteochondral defects ≥10 mm receive transplantation of an osteochondral allograft. Subjects with femoral condyle osteochondral defects <10 mm receive microfracture treatment to promote cartilage repair.	Withdrawn
NCT07312175	3D-Printed Grafts for Knee Cartilage Repair	This intervention involves 3D printing-assisted implantation of autologous periosteum and bone graft combined with platelet-rich plasma (PRP) to repair knee cartilage injuries.	Active, not recruiting
NCT02430558	Second Line Treatment of Knee Osteochondral Lesion with Treated Osteochondral Graft	This intervention involves implantation of a decellularized, freeze-dried, and irradiated osteochondral allograft to repair cartilage and bone defects.	Terminated
NCT01477008	BiPhasic Cartilage Repair Implant (BiCRI) IDE Clinical Trial - Taiwan	This intervention involves implantation of the Biphasic Cartilage Repair Implant (BiCRI) to treat chondral and osteochondral lesions, with outcomes compared to standard marrow stimulation techniques.	Completed
NCT02423629	Agili-C™ Implant Performance Evaluation in the Repair of Cartilage and Osteochondral Defects	This intervention involves implantation of the Agili-C™ scaffold, a biphasic, porous, resorbable implant designed to support regeneration of articular cartilage and osteochondral defects.	Completed
NCT01409447	Repair of Articular Osteochondral Defect	This intervention involves implantation of an autologous chondrocyte–laden biphasic cylindrical plug, composed of DL-poly-lactide-co-glycolide for the cartilage phase and β-	Recruiting

		tricalcium phosphate for the bone phase, to repair osteochondral defects.	
NCT01410136	Chondrofix Osteochondral Allograft Prospective Study	This intervention involves implantation of Chondrofix Osteochondral Allograft, an allogeneic, cylindrical, decellularized graft composed of hyaline cartilage and bone, for the repair of osteochondral lesions.	Terminated
NCT05685316	COPLA® Cartilage Implant Pilot Clinical Trial	This intervention involves implantation of the COPLA® cartilage device in combination with bone marrow stimulation during routine knee cartilage repair surgery.	Active, not recruiting
NCT02503228	Clinical Registry Assessment of the Missouri Osteochondral Allograft Preservation System - MOPS	This intervention involves implantation of Missouri Osteochondral Preservation System (MOPS) grafts for the repair of osteochondral defects.	Enrolling by invitation
NCT07332182	MaioRegen Prime Study for the Treatment of Deep Osteochondral Lesion of the Knee (OSTEOCONFIRM)	This intervention involves implantation of MaioRegen Prime, a three-layered biomimetic osteochondral scaffold, to repair osteochondral lesions of the knee.	Not yet recruiting
NCT02345564	Clinical and Radiological Results of Osteochondral Repair Using MaioRegen in Knee and Ankle Surgery	This intervention involves implantation of MaioRegen Fleece into the osteochondral lesion.	Active, not recruiting
NCT05332288	Triphasic Osteochondral Scaffold for the Treatment of the OCD of the Knee: Observational Study (MAIOCD)	This intervention involves implantation of osteochondral scaffolds using specialized surgical instrumentation.	Recruiting
NCT01183637	Evaluation of an Acellular Osteochondral Graft for Cartilage LEsions Pilot Trial (EAGLE Pilot)	This intervention involves implantation of the Kensey Nash Corp. Cartilage Repair Device, a bioresorbable scaffold designed to support repair of knee cartilage and subchondral bone.	Terminated
NCT05924763	The Clinical Outcome of BiCRI in the Treatment of Chondral and Osteochondral Lesions	This intervention involves implantation of the Biphasic Cartilage Repair Implant (BiCRI).	Completed

NCT02309957	EAGLE European Post Market Study (EAGLE)	This intervention involves implantation of BioMatrix CRD™, a sterile, biphasic, bioresorbable scaffold, to support the repair of cartilage and subchondral bone.	Completed
NCT03299959	Agili-C™ Implant Performance Evaluation	This intervention involves implantation of the Agili-C™ scaffold.	Completed
NCT01471236	Evaluation of the Agili-C Biphasic Implant in the Knee Joint	This intervention involves implantation of the Agili-C™ scaffold.	Completed
NCT00984594	Evaluation of a Composite Cancellous and Demineralized Bone Plug (CR-Plug) for Repair of Knee Osteochondral Defects	This intervention involves implantation of a human bone–derived plug, called "CR-Plug," to repair osteochondral defects.	Terminated
NCT06915233	A Study of MACI in Patients Aged 17 to 65 Years with Symptomatic Chondral or Osteochondral Defects of the Ankle (MASCOT)	This intervention involves MACI, consisting of autologous cultured chondrocytes seeded onto a porcine collagen membrane, for the repair of cartilage defects, with outcomes compared with standard bone marrow stimulation.	Recruiting
NCT03588975	A Study of MACI in Patients Aged 10 to 17 Years with Symptomatic Chondral or Osteochondral Defects of the Knee (PEAK)	This intervention involves MACI.	Recruiting
NCT01290991	A Study to Evaluate the Safety of Augment™ Bone Graft	This intervention involves implantation of Augment Bone Graft in combination with allograft to repair osteochondral defects of the knee. Augment Bone Graft is a biocompatible scaffold composed of beta-tricalcium phosphate and recombinant human platelet-derived growth factor.	Completed
NCT00821873	Evaluation of the CR Plug for Repair of Defects Created at the Harvest Site from an Autograft in the Knee	This intervention involves implantation of CR-Plug.	Completed

NCT00793104	Evaluation of the CR Plug (Allograft) for the Treatment of a Cartilage Injury in the Knee	This intervention involves implantation of CR-Plug.	Terminated
NCT04000659	Episealer® Knee System IDE Clinical Study	This intervention involves implantation of the Episealer Knee System, an endoprosthetic resurfacing implant, for the treatment of knee cartilage defects, with outcomes compared to microfracture.	Active, not recruiting
NCT06238947	Long-term Outcome Evaluation of Patients Undergoing Autologous Chondrocyte Transplantation Delivered on Biomaterial	This intervention involves implantation of autologous chondrocytes delivered on the Hyalograft C biomaterial scaffold (Fidia, Abano Terme) for the treatment of cartilage defects.	Not yet recruiting
NCT01473199	BioPoly RS Knee Registry Study for Cartilage Defect Replacement	This intervention involves surgical implantation of the BioPoly RS partial resurfacing knee implant.	Completed
NCT03262909	Pivotal Study to Evaluate the Safety and Efficacy of GelrinC for Treatment of Cartilage Defects (SAGE)	This intervention involves application of GelrinC, a biodegradable, acellular hydrogel composed of polyethylene glycol diacrylate and denatured human fibrinogen, into focal cartilage lesions immediately after microfracture.	Active, not recruiting
